# Nitric Oxide Protects against Infection-Induced Neuroinflammation by Preserving the Stability of the Blood-Brain Barrier

**DOI:** 10.1371/journal.ppat.1005442

**Published:** 2016-02-25

**Authors:** Gabriela C. Olivera, Xiaoyuan Ren, Suman K. Vodnala, Jun Lu, Lucia Coppo, Chaniya Leepiyasakulchai, Arne Holmgren, Krister Kristensson, Martin E. Rottenberg

**Affiliations:** 1 Department of Microbiology, Tumor and Cell Biology, Karolinska Institutet, Stockholm, Sweden; 2 Department of Medical Biochemistry and Biophysics, Karolinska Institutet, Stockholm, Sweden; 3 Department of Neuroscience, Karolinska Institutet, Stockholm, Sweden; University of Massachusetts Amherst, UNITED STATES

## Abstract

Nitric oxide (NO) generated by inducible NO synthase (iNOS) is critical for defense against intracellular pathogens but may mediate inflammatory tissue damage. To elucidate the role of iNOS in neuroinflammation, infections with encephalitogenic *Trypanosoma brucei* parasites were compared in *inos* ^*-/-*^ and wild-type mice. *Inos* ^*-/-*^ mice showed enhanced brain invasion by parasites and T cells, and elevated protein permeability of cerebral vessels, but similar parasitemia levels. Trypanosome infection stimulated T cell- and TNF-mediated iNOS expression in perivascular macrophages. NO nitrosylated and inactivated pro-inflammatory molecules such as NF-κΒp65, and reduced TNF expression and signalling. iNOS-derived NO hampered both TNF- and T cell-mediated parasite brain invasion. In *inos* ^*-/-*^ mice, TNF stimulated MMP, including MMP9 activity that increased cerebral vessel permeability. Thus, iNOS-generated NO by perivascular macrophages, strategically located at sites of leukocyte brain penetration, can serve as a negative feed-back regulator that prevents unlimited influx of inflammatory cells by restoring the integrity of the blood-brain barrier.

## Author Summary

Inflammatory responses can lead to harmful effects on the brain during many chronic parasitic infections, including those with African trypanosomes. *T*. *brucei*, the causative agent of African trypanosomiasis, that traverse the blood-brain barrier (BBB) to invade the brain, where, together with inflammatory infiltrates, they likely contribute to the neurologic disturbances of the disease. High levels of nitric oxide (NO) released by the inducible NO synthase (iNOS) are critical for defense against parasites, but also mediate inflammatory tissue damage. Using a mouse model of African trypanosomiasis, we uncovered an unexpected role of NO, preserving the integrity of the BBB and limiting the neuroinvasion of leukocytes and parasites, rather than mediating brain damage or killing of trypanosomes. iNOS-derived NO, nitrosylates molecules such as pro-inflammatory transcription factors. iNOS hampered both TNF- and T cell-mediated parasite and leukocyte brain invasion and passage of serum proteins across the BBB. In *inos* ^*-/-*^ mice, exacerbated TNF secretion and signalling increased MMP9 activity that mediates cerebral vascular permeability. Thus, NO is crucial for maintenance of the integrity of the cerebral vessels and serves as a feed-back regulator by inhibiting leukocyte brain penetration during *T*. *brucei* infection. Consequently, therapies could target iNOS to reduce tissue damage during neuroinflammation.

## Introduction

Nitric oxide (NO) is a gaseous molecule synthesised from l-arginine by three isoforms of the enzyme nitric oxide synthase (NOS). In the brain, NO acts as a neurotransmitter and is a component of the signalling pathways that operate between cerebral blood vessels, neurons and glial cells. The inducible NOS (iNOS) is expressed in macrophages and glial cells in response to pro-inflammatory cytokines such as IFN-γ or TNF. iNOS can produce a large amount (100–1000 times greater) of NO in relation to the other two isoforms, the endothelial NOS (eNOS) and the neuronal NOS (nNOS) [1]. After induction, iNOS continuously produces NO until the enzyme is degraded. iNOS-produced NO is a key cytotoxic weapon for the destruction of intracellular bacteria and protozoa but is also a major mediator of tissue damage. Reflecting an inflammatory component iNOS is expressed in the brain in pathological conditions such as ischemia and trauma, in which it has been suggested to contribute to increased permeability of the blood-brain barrier (BBB) and lesions in the nervous tissue [2,3]. In contrast to its role in neuroinflammation, iNOS-derived NO, similar to eNOS-derived NO, has been shown to confer protection in a model of shock, regulating the vascular tone and the blood flow in tissues [4].

The primary role of neuroinflammation is to protect the nervous system from attacks by infectious agents. However, inflammatory molecules released to prevent neuroinvasion may be detrimental to the integrity of the physical barriers and to brain functions. Thus neuroinflammation may be protective or harmful during infections. Here we analysed the consequences of iNOS-derived NO in the outcome of a parasite infection of the brain.

Infection with subspecies of the extracellular parasite *Trypanosoma brucei* (*T*.*b*.) causes human and animal African trypanosomiasis. During the early stage of infection the parasites overrun the hemolymphatic system, while during the late meningo-encephalitic stage severe signs of nervous system involvement are observed [5]. In a mouse model, the subspecies *T*.*b*. *brucei* cross the BBB at a late stage of disease and enter the brain parenchyma. Within the CNS, leukocyte infiltrates and activation of resident glial cells likely contribute to the nervous system dysfunctions that are characteristic of the disease, which in humans is also called “sleeping sickness” [6]. The brain invasion by trypanosomes is a complex process regulated by different host-derived factors. We have previously observed that brain invasion by *T*.*b*. *brucei* and T cells depends on the secretion of IFN-γ, the IFN-γ-dependent chemokine CXCL10 and on TNF [7–9].

Human and animal infections with *T*.*b*. demonstrate that NO production is elevated across species [10–12]. However, the role of NO during African trypanosomiasis is controversial. Studies on a *T*. *congolense* model indicate that NO has a protective role [13,14], while in a *T*.*b*. model NO instead induces anaemia [15] or contributes to increased parasite levels through down regulation of cellular immune responses [16]. Others have shown that the NO synthesized in *T*.*b*.-infected mice lacks trypanocidal activity **in vivo** [16–18]. While there is consensus that NO generation is stimulated by infection, the role of iNOS-generated NO during the infection and specifically in the brain at the encephalitic stage is still unclear.

In the present study, we investigated the role of iNOS-derived NO in neuroinflammation induced by infection with *T*.*b*. *brucei* in mice. Our results indicate that iNOS plays a critical but unexpected anti-inflammatory role by hampering a T cell and TNF-mediated penetration of *T*.*b*. *brucei* and leukocytes into the brain and maintaining the integrity of the cerebral vessels.

## Results

### iNOS-derived NO impedes trypanosome and T cell invasion into the brain

**Fig 1:**
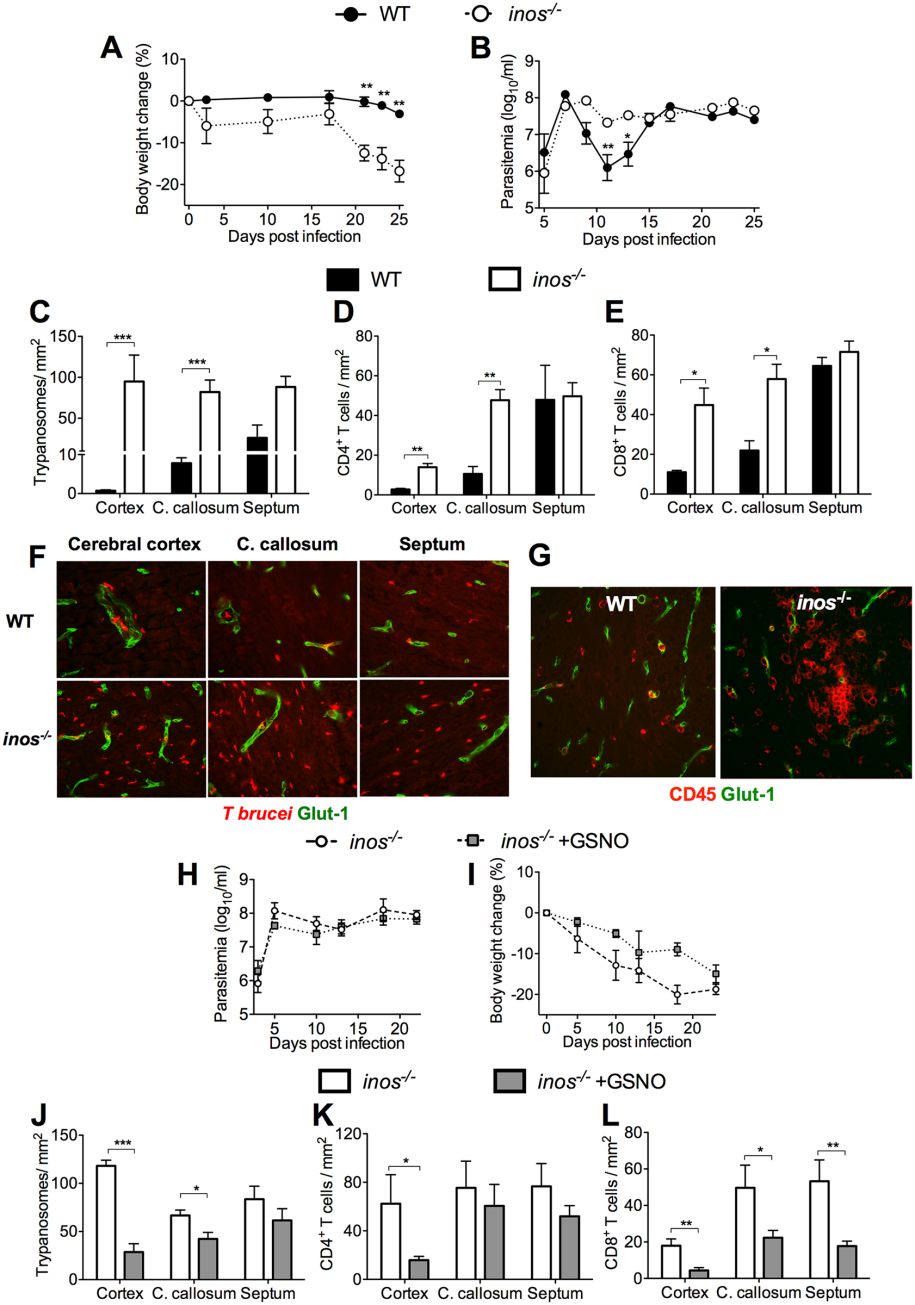
iNOS-derived NO reduces *T*.*b*. *brucei* and leukocyte penetration into the brain. (A-B) Body weight and parasitemia of WT and *inos* ^*-/-*^ mice infected i.p. with 2x10^3^ *T*.*b*. *brucei*. Each point represents the mean log_10_ parasites per ml ± SEM (n = 9 to 10 per group). Statistically significant differences in comparison with infected WT animal (*p< 0.05, **p< 0.01 two-way ANOVA). The body weights were standardized with respect to the mean of the same group before infection. One out of three independent experiments is depicted. (C-F) The mean number of *T*.*b*. *brucei* (C), and CD4+ (D), and CD8+ (E) T cells per mm^2^ ± SEM from 6 animals per group is depicted. (F) Representative immunofluorescence images show *T*.*b*. *brucei* and cerebral endothelial cells in cerebral regions of WT and *inos* ^*-/-*^ mice 25 dpi. A representative of three similar independent experiments is shown. Statistically significant differences in comparison to WT mice at the same dpi: (*p<0.05, **p<0.01 and ***p<0.001 unpaired Student’s *t* test). (G) Representative fluorescent staining of CD45+ leukocytes and cerebral endothelial cells (Glut-1) of WT and *inos* ^*-/-*^ mice 25 dpi. (H, I) Parasitemia (H) and weight (I) of *inos* ^*-/-*^ mice infected i.p. with *T*. *brucei* and treated or not daily with 3.5 mg GSNO i.p. starting at 5 dpi. (J-L) The mean numbers of *T*.*b*. *brucei* (J), CD4+ (K) and CD8+ (L) cells per mm^2^ in the brain of mice (n = 6 per group) sacrificed 23 dpi is shown (*p<0.05, **p<0.01 and ***p<0.001 unpaired Student’s *t* test).

The role of iNOS-derived NO in the outcome of infection with *T*.*b*. *brucei* was first studied. *T*.*b*. *brucei*-infected *inos* ^-/-^ mice showed a significant loss of body weight compared to WT controls (Fig 1A), and all *inos* ^*-/-*^ mice died at earlier than controls (S1A Fig). After an initial peak of parasitemia WT, but not *inos* ^*-/-*^, mice controlled blood parasite levels. On the other hand *inos* ^*-/-*^ and WT mice showed similar parasitemia levels at all time points later than 15 days post infection (dpi) (Fig 1B).

Whether trypanosomes are more sensitive to toxicity of NO as compared to mammalian cells was then tested. We found that *T*.*b*. *brucei* and various mammalian cell lines displayed similar susceptibility to the NO-donors SNAP and GSNO (S1 Table).

The role of iNOS expression in the brain invasion by trypanosomes and leukocytes was then studied. Unexpectedly, significantly higher numbers of parasites and CD4^+^ and CD8^+^ T cells were found in the cerebral cortex and corpus callosum of *inos* ^*-/-*^ when compared to WT mice (Fig 1C–1F). Perivascular and intraparenchymal clusters of CD45^+^ leukocytes were seen in the brain of *T*.*b*. *brucei-*infected *inos* ^*-/-*^ mice, which were less frequent in WT mice (Fig 1G). Parasites localized within blood vessels in brains of *inos* ^*-/-*^ mice at 10 and 16 dpi, indicating that the enhanced *T*. *brucei* density in the brain parenchyma at later times point after infection, is not due to an earlier brain invasion (S1B Fig).

We tested whether the administration of the NO donor GSNO could diminish *T*.*b*. *brucei* and T cell numbers in the brain of *inos* ^*-/-*^ mice. Infected *inos* ^*-/-*^ mice inoculated daily with GSNO showed similar parasitemia levels as untreated controls (Fig 1H), but a delay in body weight loss as well as lower parasite and T cell numbers in the brain compared to untreated mice (Fig 1I–1L). Thus, exogenous NO administration reverses in part the increased brain invasion observed in *T*.*b*. *brucei*-infected *inos* ^*-/-*^ mice.

### Increased BBB permeability in *T*.*b*. *brucei*-infected *inos* ^*-/-*^ mice

**Fig 2:**
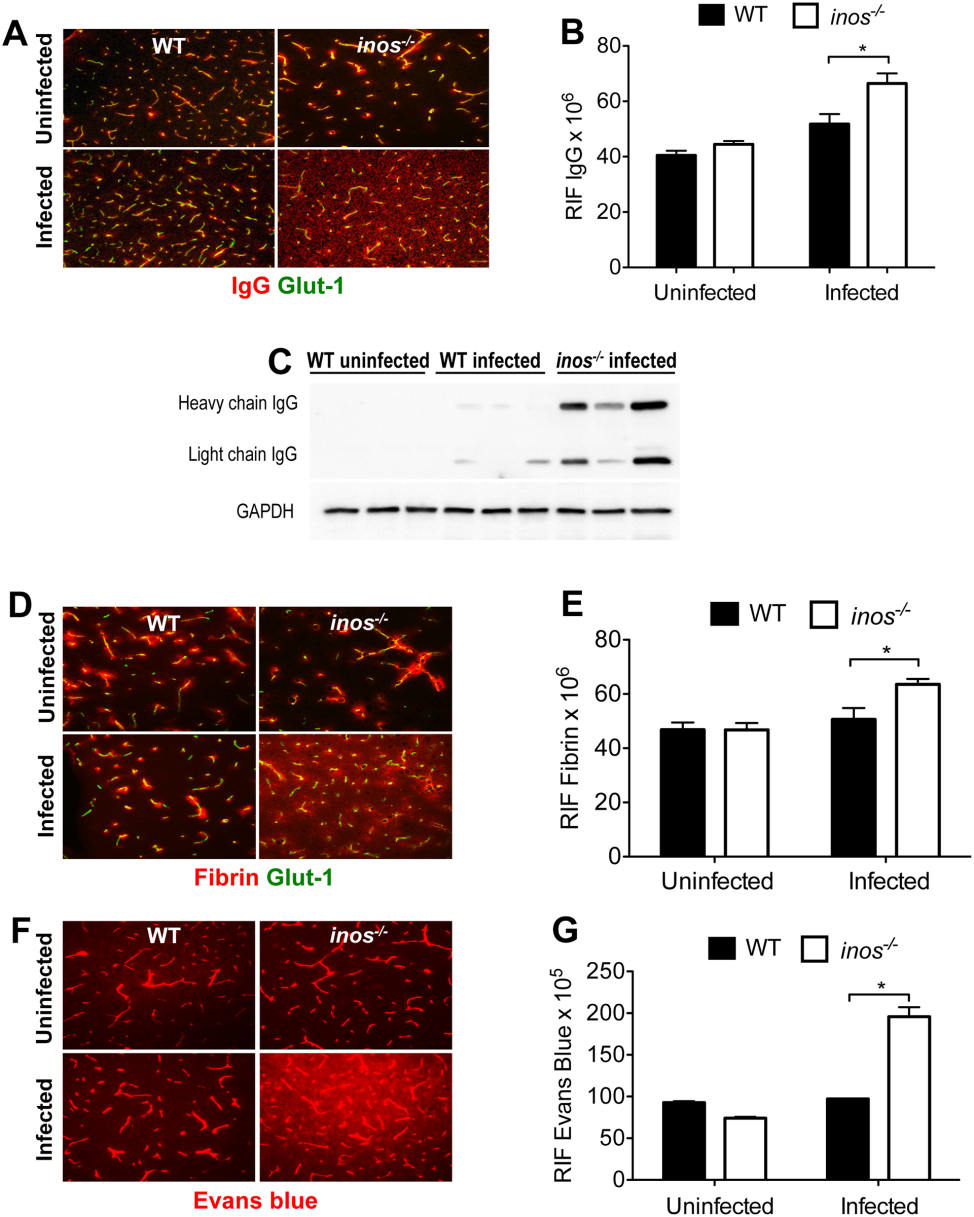
iNOS expression is associated with integrity of the BBB during infection with *T*.*b*. *brucei*. (A, D, F) IgG (A) and fibrin (D) immunolabeling and Evan’s blue extravasation (F) in the brains of WT and *inos* ^*-/-*^ mice 23 dpi with *T*.*b*. *brucei*. (B, E, G) The mean relative integrated fluorescence densities (RIF) of IgG (B), fibrin (E) or EB (G) ± SEM in the brain parenchyma from at least 3 sections per brain and 4 animals per group are depicted. (C) IgG was also detected by Western Blot in brain lysates of *inos* ^*-/-*^ mice 25 dpi with *T*.*b*. *brucei* but not in infected or uninfected WT mice. Differences between WT and *inos* ^*-/-*^ mice are significant (*p<0.05 unpaired Student’s *t* test).

We then examined if alteration in the permeability of the BBB could underlie the highly increased parasite and inflammatory cell invasion of the brain during the late stage of *T*.*b*. *brucei* infection in *inos* ^*-/-*^ mice. *T*.*b*. *brucei-*infected *inos* ^*-/-*^ mice showed increased deposits of IgG and fibrin in the brain parenchyma while vascular leakage was not obvious in infected WT mice or in non-infected *inos* ^*-/-*^ or WT controls, in which IgG and fibrin remained intravascularly (Fig 2A, 2B, 2D and 2E). IgG was also found in brain lysates from infected *inos* ^*-/-*^ mice, but neither in infected WT nor in non-infected controls (Fig 2C). The increased vascular permeability in the brain of *inos* ^*-/-*^ mice was confirmed in experiments using Evans blue as a tracer. Evans blue was found in the brain parenchyma of *T*.*b*. *brucei*-infected *inos* ^*-/-*^ mice, but not in WT mice (Fig 2F and 2G).

Complexes of endothelial tight junction molecules contribute to the impermeability of the BBB. *T*.*b*. *brucei*-infected WT and *inos* ^*-/-*^ mice showed similar immunolabelling of the junctional components claudin-5, zona occludens-1 (ZO-1) and occludin (S2A Fig). The junctional markers were distributed in a pattern consistent with continuous junction complexes in brain capillaries in both mutants and controls. In line with this, the expression of *claudin-5* and *occludin* mRNA in the brain of *inos* ^*-/-*^ and WT infected or control mice was similar (S2B and S2C Fig). This suggest a functional change in interactions between proteins that regulate permeability of the tight junctions rather than their loss [19].

Infected *inos* ^*-/-*^ mice showed a more pronounced astrocytic reaction, as observed by increased labelling of the glial fibrillary acidic protein (GFAP), than WT-infected mice (S2D Fig). *Iba-1* mRNA levels, a marker of microglia activation, was also increased in brains from *inos* ^*-/-*^ as compared to WT mice (S2E Fig). *Inos* ^*-/-*^ but not WT infected mice showed minor signs of axonal degeneration as visualized by labelling with β-APP, an antibody that binds to degenerating neurons (S2F Fig). Finally we investigated if pericytes, a main constituent of the BBB contributing to its integrity [20], were diminished in *inos* ^*-/-*^ mice. The density of pericytes along the brain blood vessels in *inos* ^*-/-*^ and WT mice as stained for chondroitin sulphate proteoglycan was similar (S2G Fig).

Altogether, the BBB of *T*.*b*. *brucei*-infected *inos* ^*-/-*^ mice show an enhanced permeability to proteins, which is accompanied by severe inflammatory changes, astrogliosis, increased microglial activation and mild neuronal degeneration.

### Infection with *T*.*b*. *brucei* stimulates the expression of iNOS in macrophages

**Fig 3:**
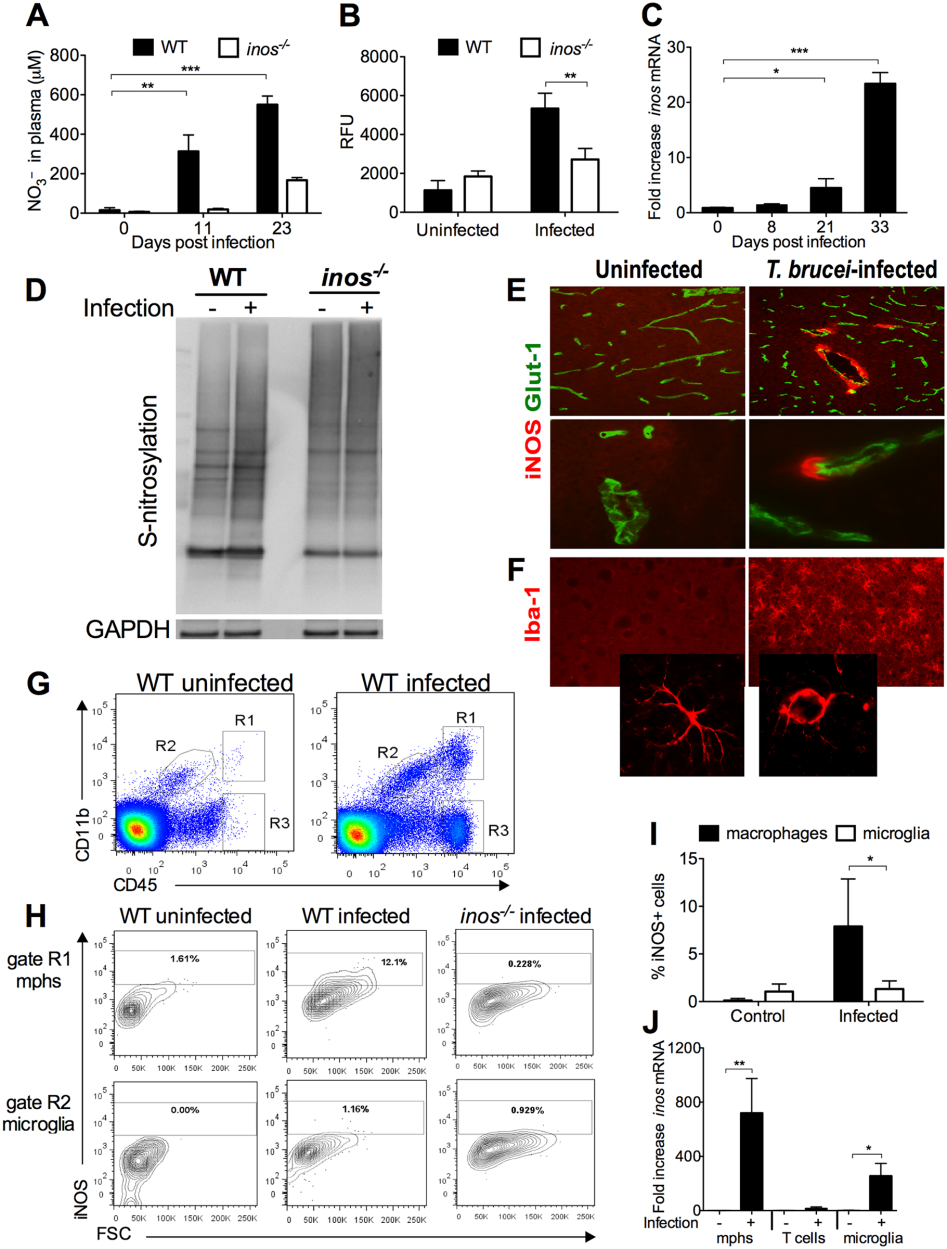
iNOS is expressed by perivascular macrophages in the brain during infection with *T*.*b*. *brucei*. (A) Concentration of NO_3_ in plasma as measured by Griess assay after nitrate reductase reaction. The mean NO_3_ concentration ± SEM in the plasma of infected mice (n = 5 per time point) is depicted. Differences with uninfected controls are significant (**p<0.01, ***p<0.001, unpaired Student’s *t* test). (B) Levels of S-nitrosylated molecules in plasma as measured using the 2,3-diaminonapthalene (DAN) assay. The mean relative fluorescence units (RFU) ± SEM in WT and *inos* ^*-/-*^ infected and control animals are indicated. Differences with uninfected control and *inos* ^*-/—*^infected mice are significant (**p<0.01, unpaired Student’s t test). (C) The accumulation of *inos* or *hprt* transcripts in brains sampled at various dpi with *T*.*b*. *brucei* was measured by real time PCR. The mean fold *inos* mRNA increase ± SEM in brains from infected mice (n ≥ 5 per group) is depicted. Differences with controls are significant (*p<0.05; ***p<0.001, unpaired Student’s *t* test). (D) Levels of S-nitrosylated proteins in brain lysates from *T*.*b*. *brucei* infected mice were measured by the biotin switch assay as described in the supplemental methods (S1 Text). GAPDH was used as a loading control. Similar results were obtained in 3 independent experiments. (E) iNOS labelling in the brain of WT mice at 0 or 30 dpi with *T*.*b*. *brucei*. (F) Immunolabelling with the activated microglia marker Iba-1 in a WT mouse. (G) FACS analysis of CD45^high^CD11b^+^ macrophages (R1), CD45^dim^ CD11b^+^ microglia (R2) and CD45^high^ CD11b^-^ lymphocytes (R3) in the brain of WT mice was determined at 0 or 25 dpi. (H) The frequency of iNOS^+^ cells in gated populations (panel G) are depicted. Cells from *inos* ^*-/-*^ infected mice were used as negative controls. (I) The mean percentage ± SEM of iNOS^+^ CD45^high^ CD11b^+^ or CD45^dim^ CD11b^+^ (n = 4 per group) is depicted. (J) Levels of *inos* transcripts in sorted CD45^high^CD11b^+^, CD45^dim^CD11b^+^ and CD45^high^ CD11b^-^ populations are shown. Three mice were pooled for each determination and at least 4 independent determinations were performed.

Since iNOS-derived NO abrogated changes in BBB permeability, the kinetics of NO generation during *T*.*b*. *brucei* infection and the localization of iNOS expressing cells in the brain were then analysed. Nitrates were elevated in the sera from infected mice as compared to uninfected controls (Fig 3A). S-nitrosylation, the covalent binding of NO to the thiol side chain of cysteine, is an important post-translational regulatory mechanism of most main classes of proteins. The S-nitrosothiol levels were elevated in the sera of *T*.*b*. *brucei-*infected WT, but not in that of *inos* ^*-/-*^ mice (Fig 3B).

Brains from *T*.*b*. *brucei*-infected mice showed increased *inos* mRNA levels as compared to uninfected controls (Fig 3C). S-nitrosylated proteins were elevated in brain lysates of *T*.*b*. *brucei*-infected as compared to uninfected mice using a biotin switch assay. On the other hand, brains from infected and uninfected *inos* ^*-/-*^ mice showed similar S-nitrosylation levels (Fig 3D).

The cellular source of iNOS was investigated by immunolabelling. iNOS expressing cells were found in cells in the leptomeninges, the choroid plexus and in cells around cerebral vessels in both the grey and the white matter in *T*.*b*. *brucei*-infected mice, but not in uninfected controls (Fig 3E). Activated Iba^+^ microglial cells were increased after infection. However, the dense distribution of Iba1^+^ cells suggests that most of them do not express iNOS (Fig 3F).

CD45^high^CD11b^high^ inflammatory cells were observed in brain cell suspensions from infected but not from non-infected animals. Most of these cells were Ly6C+ and Ly6G^neg^ suggesting these are enriched in inflammatory monocyte/ macrophages, but not in granulocytes (S3A, S3C and S3D Fig) [21]. CD45^dim^CD11b^high^ microglial cells were observed in both infected and non-infected brains (Fig 3G). The majority of the CD11b^neg^CD45^high^ cells were CD3^+^ (S3B Fig). Confirming the results from the tissue immunolabelling, iNOS^+^ macrophages were detected in suspensions of brain cells of infected animals. In contrast, microglial cells showed no iNOS labelling (Fig 3H and 3I), although they showed a change from a ramified resting form to an ameboid morphology, characteristic of activated microglia, after *T*.*b*. *brucei* infection (Fig 3F). Similarly, CD45^high^CD11b^high^ cells from brains from infected mice had higher levels of *inos* mRNA than microglia- or T cell-enriched populations (Fig 3J).

### iNOS-derived NO hampers TNF expression in the brain of *T*.*b*. *brucei* infected mice

**Fig 4:**
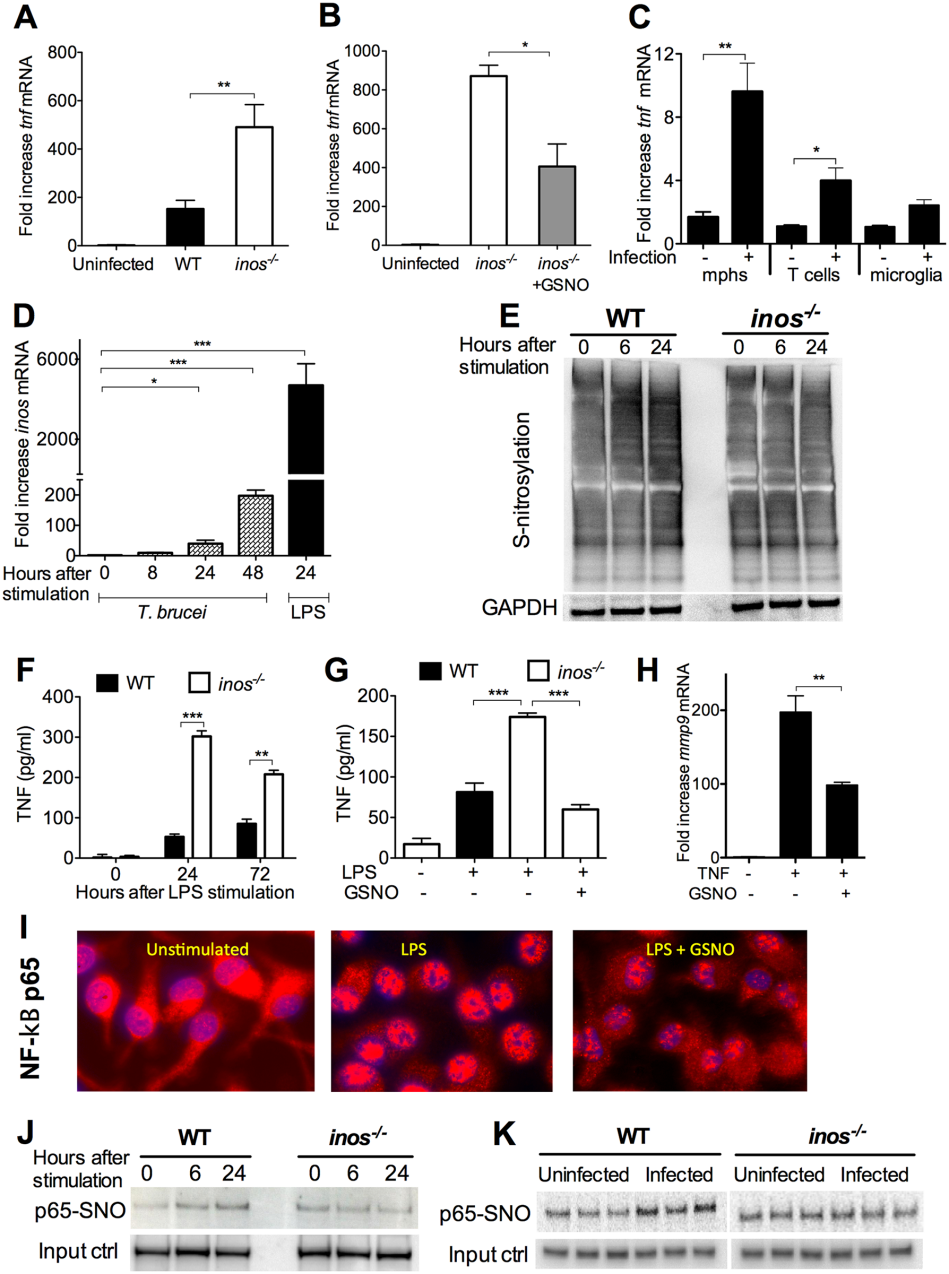
*Tnf* mRNA is increased in brains of *inos* ^*-/-*^ mice infected with *T*.*b*. *brucei*. (A) The total RNA was extracted from brains of WT and *inos* ^*-/-*^ *T*.*b*. *brucei-*infected mice sacrificed 23 dpi or uninfected controls. (B) In other sets of experiments RNA was also isolated from infected *inos* ^*-/-*^ mice treated daily with GSNO starting 5 dpi. The mean fold *tnf* mRNA increase ± SEM in brains from infected mice (n ≥ 4 per group) was calculated by real time PCR. (C) *Tnf* mRNA in sorted macrophage, microglia and T cell-enriched brain cell populations of *T*.*b*. *brucei*-infected and uninfected WT mice. Differences with controls are significant (*p<0.05, **p<0.01 Student’s *t* test). (D) *Inos* mRNA levels were measured in triplicate cultures of WT BMM at different time points after stimulation with *T*.*b*. *brucei* lysates (MOI 5:1) or 1 μg/ ml LPS. Differences with controls are significant (*p<0.05, ***p<0.001 Student t test). (E) Nitrosylated proteins in lysates obtained before and after LPS-stimulation of WT and *inos* ^*-/-*^ BMM were detected by the biotin switch assay. GAPDH was used as a loading control. One of 3 independent experiments is shown. (F, G) The concentration of TNF in supernatants of LPS-stimulated BMM treated or not with 200 μM GSNO were measured by ELISA. The mean TNF titters in triplicate cultures ± SEM are depicted. Differences with LPS-stimulated *inos* ^*-/-*^ BMM are significant (*p<0.05, ***p<0.001 Student’s *t* test). (H) The levels of *mmp9* mRNA were determined in BMM lysates 24 h after incubation with 100 ng/ ml TNF in presence or not of GSNO. Differences are significant (**p<0.01 Student’s *t* test). (I) Immunofluorescence of NF-κΒ p65 (red) and DAPI in LPS-stimulated BMM treated or not with GSNO. (J, K) Detection of NF-κΒ p65 in lysates of nitrosylated proteins from LPS-stimulated BMM (J) and of brains of *T*.*b*. *brucei*-infected mice (K). The biotin switch reaction was performed on lysates, which were then immunoprecipitated with neutroavidin agarose. NF-κΒ p65 in the IP was then detected in a WB (see S1 Text). The levels of total NF-κΒp65 were used as loading controls.

The mechanisms underlying the iNOS-mediated impediment of *T*.*b*. *brucei* and leukocyte invasion of the brain were studied. Brains from *T*.*b*. *brucei*-infected *inos* ^*-/-*^ mice contained higher *tnf* mRNA levels than controls (Fig 4A). The level of *tnf* mRNA was reduced in infected *inos* ^*-/-*^ mice treated with GSNO as compared to untreated controls (Fig 4B). Vascular endothelial cells respond to TNF by undergoing a number of pro-inflammatory changes including an increased expression of adhesion molecules. The transcript levels of TNF-regulated *vcam1* and *icam1* but not *e-selectin*, were also increased in the brains of *T*.*b*. *brucei*-infected *inos* ^*-/-*^ mice compared to WT controls (S4A, S4C and S4E Fig). However, similar levels of *vcam1* and *icam1* mRNA were found after GSNO treatment (S4B and S4D Fig). Brains from *T*.*b*. *brucei-*infected WT and *inos* ^*-/-*^ mice showed similar levels of *il1b*, *il6* and *il17a* transcripts (S4F–S4H Fig).

Recruitment of leukocytes into the brain during infection with *T*.*b*. *brucei* is chemokine-dependent. The levels of *ccl2*, *cxcl9* and *cxcl10* transcripts were increased in brains of infected mice compared to controls. Furthermore the levels of *ccl2* and *cxcl10* but not *cxcl9* mRNA were augmented in brains from *inos* ^*-/-*^ mice as compared to infected WT controls (S4I–S4K Fig).

Macrophage, but not microglia, -enriched cell suspensions from the brain had increased levels of *tnf* mRNA after infection compared to uninfected controls (Fig 4C). T cells isolated from infected brains also showed higher *tnf* mRNA content than controls from uninfected animals (Fig 4C).

We then performed **in vitro** experiments with primary BMM to determine if the elevated *tnf* mRNA levels in *inos* ^*-/-*^ brains could be a consequence of the increased numbers inflammatory cells and parasites in the brain or the down-regulation of the cytokine expression by iNOS-derived NO. BMM showed increased *inos* mRNA after stimulation with LPS or parasite lysates (Fig 4D). Moreover enhanced levels of S-nitrosylated proteins were observed in WT, but not in *inos* ^*-/-*^ BMM stimulated with LPS (Fig 4E). Similar to the findings in the brains of infected *inos* ^*-/-*^ mice, TNF levels in supernatants from LPS-stimulated *inos* ^*-/-*^ BMM were higher than those from WT controls (Fig 4F), and treatment with GSNO decreased TNF levels in supernatants from LPS-stimulated BMM (Fig 4G). Importantly, GSNO suppressed not only TNF secretion but also TNF-mediated responses: Decreased levels of *mmp9* mRNA were observed when TNF-stimulated BMM were incubated with GSNO (Fig 4H).

NF-κΒ activation mediates TNF expression. Stimulation of BMM with LPS led to translocation of NF-κΒ p65 into the cell nucleus. Importantly, the nuclear translocation NF-κΒ p65 was impaired by addition of GSNO to the culture (Fig 4I). In pull down experiments NF-κΒ p65 was identified as one of the differentially nitrosylated proteins in LPS-stimulated WT BMM (Fig 4J). Nitrosylation of NF-κΒ p65 was also observed in brains from infected WT, but not *inos* ^*-/-*^ mice (Fig 4K).

Thus, incubation of BMM with LPS increases iNOS expression and nitrosylation of various intracellular proteins, including NF-κΒ p65. NO hampers NF-κΒ p65 translocation into the nucleus and TNF production in BMM as well as in the brain of infected mice.

Activation of MAPK-p38 is required for the synthesis of TNF in LPS-stimulated macrophages [49]. Contrary to the results obtained for NF-κΒ p65, neither endogenous iNOS expression nor exogenous GSNO hampered the activation MAPK p38 in BMM stimulated with LPS (S5 Fig). Thus, the NO-mediated inhibition of TNF secretion is not due to a hampered MAPKp38 activation.

### iNOS hampers TNF-mediated vascular leakage and penetration of *T*.*b*. *brucei* and T cells into the brain

**Fig 5:**
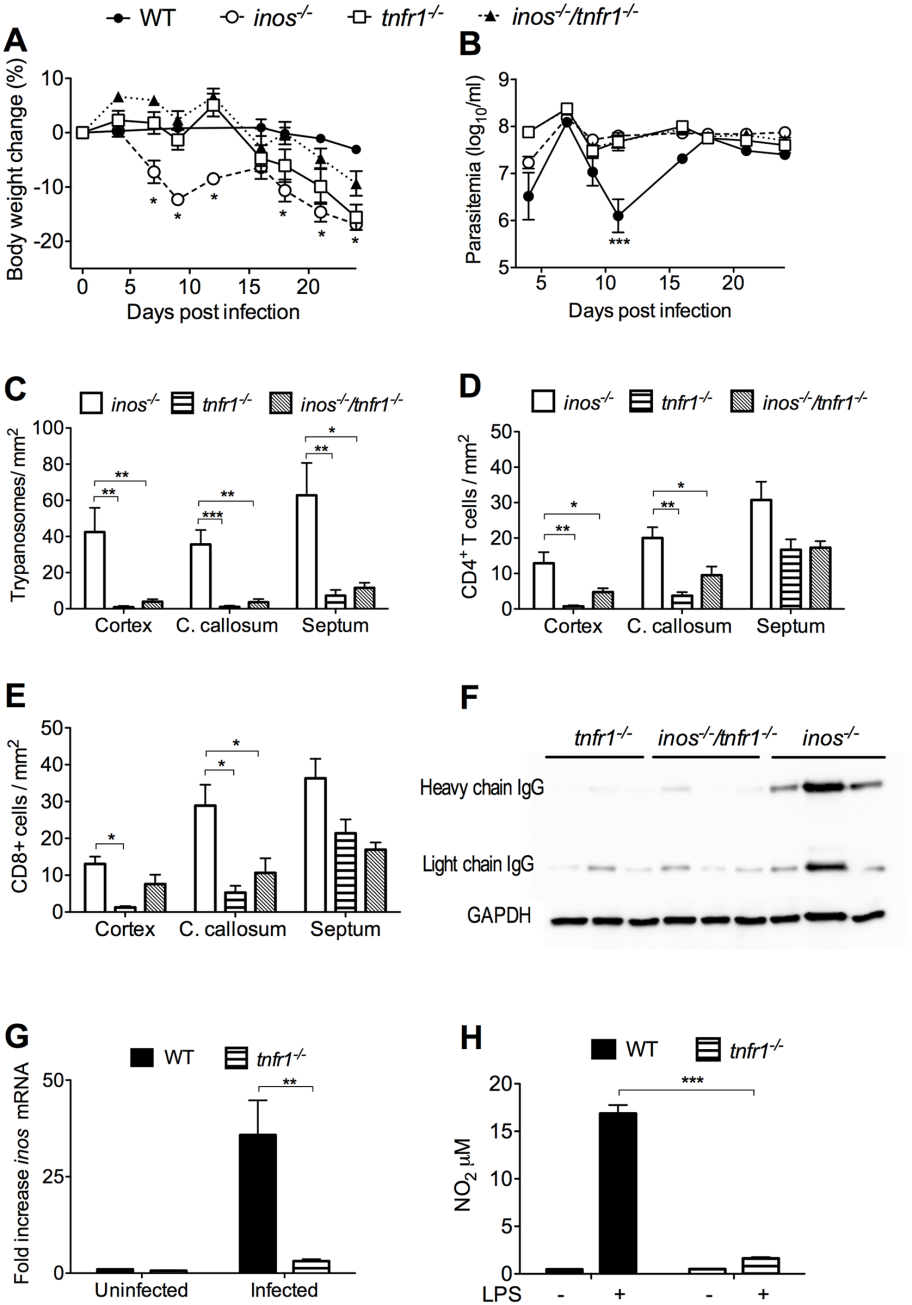
iNOS protects against TNF-mediated penetration of *T*.*b*. *brucei* into the brain. (A, B) Mean body weight and log_10_ parasites per ml of *inos* ^*-/-*^/ *tnfr1* ^*-/-*^, *tnfr1* ^*-/-*^, *inos* ^*-/-*^ and WT mice infected with *T*.*b*. *brucei* ± SEM (n = 10 per group). The body weights are relative to the weight of each group before infection. Differences with infected WT animals are significant (*p<0.05, **p< 0.01 two-way ANOVA). One of two independent experiments is depicted. (C-E) The mean number of *T*.*b*. *brucei* (C), CD4^+^ (D), CD8^+^ T cells (E) cells per mm^2^ in the cerebral regions of mice at 23 dpi ± SEM (n = 6). One out of two independent experiments is depicted. Differences with WT mice at the same dpi are significant (*p< 0.05, **p< 0.01, ***p<0.001 unpaired Student’s *t* test). (F) IgG was detected by Western blot in brain lysates of *tnfr1* ^*-/-*^, *inos* ^*-/-*^ */tnfr1* ^*-/-*^and *inos* ^*-/-*^ mice 23 dpi with *T*.*b*. *brucei*. (G) Accumulation of *inos* mRNA increase ± SEM in brains from WT and *tnfr1* ^*-/-*^ mice at 23 dpi (n ≥ 5 per group) was calculated. Differences with controls are significant (**p<0.01 Student’s *t* test). (H) The concentration of NO_2_ was measured in the 24 h supernatants of LPS-stimulated WT and *tnfr1* ^-/-^ BMM using a Griess assay. The mean NO_2_ levels ± SEM in triplicate cultures per condition are depicted. Differences with WT control are significant (***p<0.001 Student’s *t* test).

In order to study whether TNF mediates the increased permeability to proteins, leukocytes and parasites in the brain of *inos* ^*-/-*^ mice, the outcomes of the infection with *T*.*b*. *brucei* in *inos* ^*-/-*^, *tnfr1* ^-/-/-^ and *inos* ^*-/-*^/ *tnfr1* ^*-/-*^ mice were compared. All *inos* ^*-/-*^, *tnfr1* ^*-/-*^ and *inos* ^*-/-*^ */ tnfr1* ^*-/-*^ mice lost more weight compared to WT infected controls (Fig 5A). Neither *inos* ^*-/-*^, *tnfr1* ^-/-/-^ nor *inos* ^*-/-*^/ *tnfr1* ^-/-/-^ mice were able to control the parasitemia after the first week after infection, but all showed similar parasitemia levels as WT mice during the late stage of infection (Fig 5B). However, the parasite and T cells numbers in the brain of *tnfr1* ^*-/-*^ mice were strikingly reduced as compared to the *inos* ^*-/-*^ mice (Fig 5C–5E). Brain invasion of parasites and T cells was also diminished in *inos* ^*-/-*^/ *tnfr1* ^*-/-*^ mice (Fig 5C–5E).

The increased vascular permeability to IgG in the brain in infected *inos* ^*-/-*^ mice was observed neither in *tnfr1* ^-/-^ nor in *inos* ^*-/-*^/ *tnfr1* ^*-/-*^ mice (Fig 5F) indicating that in absence of TNF-R1 signalling the lack of iNOS-derived NO has no effect on the BBB permeability in this inflammatory condition.

Interestingly, *inos* mRNA levels were reduced in brains from *tnfr1* ^-/-^ mice as compared to WT controls (Fig 5G). In line with this, LPS-treated *tnfr1* ^*-/-*^ macrophages had diminished nitrite levels in culture supernatants as compared to WT controls (Fig 5H). This indicates that TNF induces iNOS-derived NO and that NO regulates TNF production.

Thus, TNF stimulates iNOS expression in brain macrophages (and in macrophages **in vitro**). On the other hand, iNOS*-*derived NO hampers a TNF-mediated cerebral vascular leakage and brain invasion of leukocytes and parasites.

### MMP9 mediates parasite and T cell penetration and vascular leakage into the brain in *T*.*b*. *brucei*-infected *inos* ^*-/-*^ mice

The mechanisms involved in TNF-mediated brain invasion of parasites and T cells in *inos* ^*-/-*^ mice were then studied. Activated matrix metalloproteinases (MMP) are known to facilitate white blood cell invasion into the brain parenchyma. Some of these enzymes regulate signalling downstream of the TNF receptor [22].

**Fig 6:**
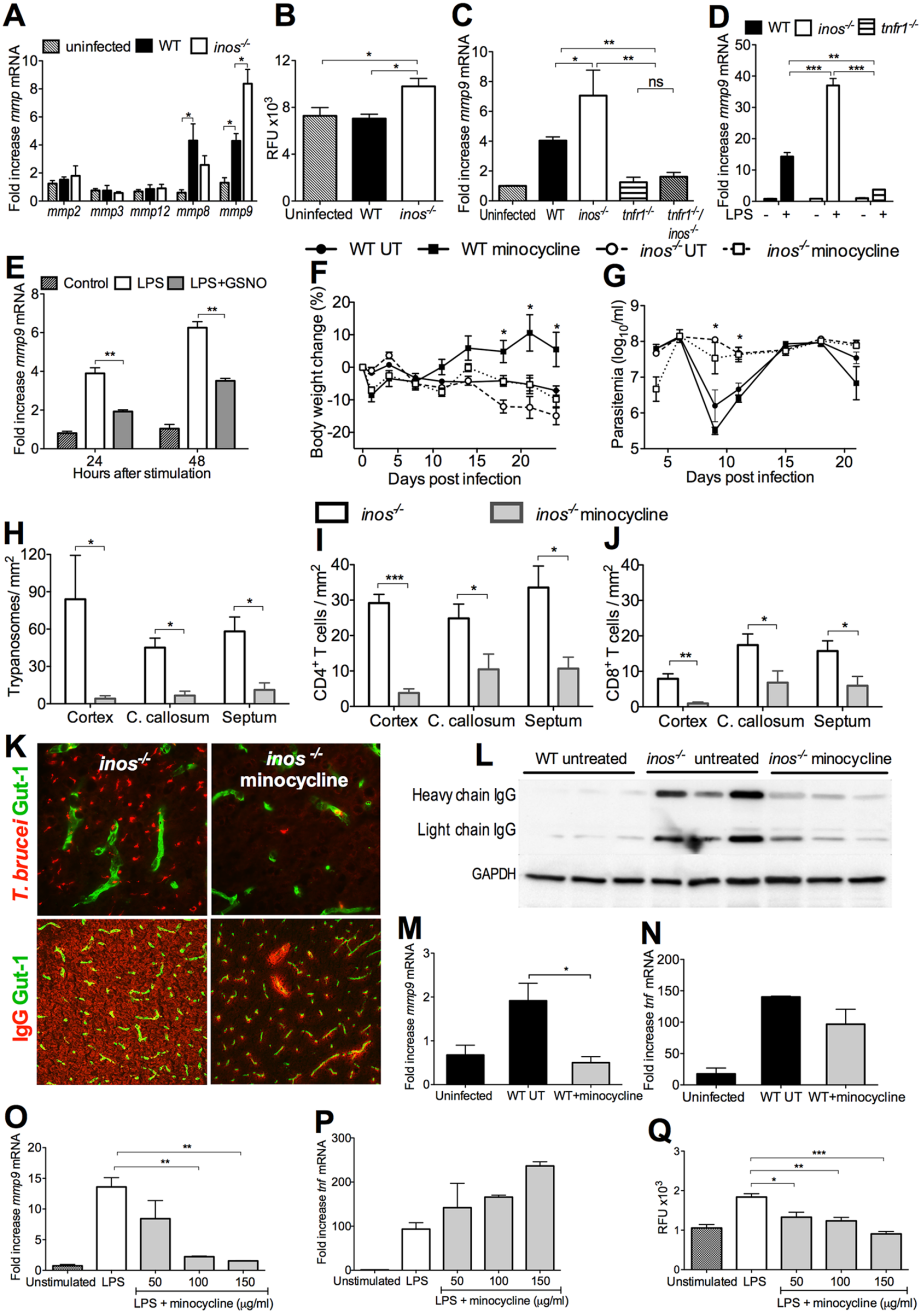
MMP9 mediates vascular leakage and parasite penetration into the brain of *T*.*b*. *brucei*-infected *inos* ^*-/-*^ mice. (A, C) The mean fold increase of *mmp* mRNA ± SEM (n ≥ 5 per group) was measured in RNA from the brain of mice 23 dpi or uninfected controls by real time PCR. One of two independent experiments is shown. Differences with controls are significant (*p<0.05, **p<0.01 Student’s t test). (B) MMP activity in brain lysates from infected mice was determined using a fluorescent MMP FRET peptide substrate. The mean relative fluorescent units (RFU) ± SEM of 4 animals per group are depicted. Differences with uninfected mice are significant (*p<0.05 Student’s t test). (D, E) RNA was obtained from LPS stimulated or control WT, *inos* ^*-/-*^ and *tnfr1* ^*-/-*^ BMM and from *inos* ^*-/-*^ BMM treated or not with GSNO. The mean fold increase of *mmp9* mRNA in triplicate cultures ± SEM is depicted. (F, G) Mean weight and log_10_ parasites per ml of WT and *inos* ^*-/-*^ mice (n = 10 per group) infected with *T*.*b*. *brucei* and treated or not i.p. with minocycline. Differences to the WT group are significant (*p<0.05 ANOVA). (H-J) The mean number of *T*.*b*. *brucei* (H), CD4^+^ (I), CD8^+^ T cells (J) cells per mm^2^ in the cerebral regions of *inos* ^*-/-*^ mice treated or not with minocycline at 23 dpi ± SEM (n = 5 per group). (K) Immunofluorescence micrograph from the cortex showing *T*.*b*. *brucei* (upper micrographs 63x) or IgG (lower 25x) at 23 dpi treated or not with minocycline. (L) IgG in brain lysates of infected *inos* ^*-/-*^ mice treated or not with minocycline was detected by Western blot. (M, N) Total RNA was extracted from the brains of *T*.*b*. *brucei-*infected WT mice treated or not with minocycline at 23 dpi. The mean fold increase of *mmp9* (M) and *tnf* (N) mRNA ± SEM in brains from infected mice (n ≥ 5 per group) was determined. Differences with controls are significant (*p<0.05 Student’s t test). (O, P) *Mmp9* and *tnf* mRNA were measured in lysates from LPS stimulated *inos* ^*-/-*^ BMM in presence of various concentrations of minocycline. Differences with untreated controls are significant (**p<0.01 Student’s t test). (Q) MMP activity in supernatants from LPS treated BMM in presence or absence of minocycline was determined using a fluorescent MMP FRET peptide substrate. The mean RFU ± SEM in triplicate cultures are depicted. Differences to untreated BMM are significant (*p<0.05, **p<0.01 ***p<0.001 Student’s t test).

Several *mmp* transcripts were previously shown to be increased in the brain during early and late stage *T*.*b*. *brucei* infection[23]. Among these, *mmp8* and *mmp9* mRNA levels were increased in the brain of mice at 23 dpi (Fig 6A). However only the *mmp9* mRNA level was further increased in the brain of infected *inos* ^*-/-*^ as compared to WT mice (Fig 6A). The MMP activity was also increased in brain lysates from *T*. *brucei-*infected *inos* ^*-/-*^ mice (Fig 6B). *Mmp9* mRNA levels were increased in CD45^high^CD11b^+^ macrophages, but not in T cells or microglia, after *T*.*b*. *brucei* infection (S6 Fig). Brains from infected *tnfr1* ^*-/-*^ and *inos* ^*-/-*^/ *tnfr1* ^*-/-*^ mice showed lower *mmp9* mRNA levels than *inos* ^*-/-*^ or WT mice (Fig 6C). Similarly *mmp9* mRNA levels were higher in LPS-stimulated *inos* ^*-/-*^ than in WT BMM (Fig 6D) and were reduced by treatment of BMM with GSNO (Fig 6E).

We have previously reported that daily administration of minocycline, a tetracycline antibiotic, impedes the penetration of leukocytes and trypanosomes into the brain parenchyma of *T*.*b*. *brucei*-infected WT mice [24]. Since minocycline can inhibit the expression and activation of MMPs [25], we studied its effect on the brain invasion in *inos* ^*-/-*^ mice. The body weight loss of both *T*.*b*. *brucei-*infected WT and *inos* ^*-/-*^ mice was reduced by daily administration of minocycline (Fig 6F), while the parasitemia levels were similar in minocycline treated and untreated groups (Fig 6G). The number of parasites and T cells in the brain of *inos* ^*-/-*^ mice treated with minocycline were reduced when compared with the untreated controls (Fig 6H–6K). Moreover, treatment with minocycline resulted in a reduced IgG in the brain parenchyma of *inos* ^*-/-*^ infected mice (Fig 6K and 6L).

Brains from minocycline-treated, infected WT mice showed decreased *mmp9*, but not *tnf* mRNA levels compared to untreated infected controls (Fig 6M and 6N). In line with this, reduced *mmp9*, but not *tnf* mRNA levels were observed after co-incubation of LPS-stimulated BMM with minocycline (Fig 6O and 6P). Minocycline treatment also reduced MMP activity of LPS-stimulated BMMs (Fig 6Q). Thus, minocycline does not alter TNF, but reduces MMP9 levels and has a striking protective effect on neuroinflammation in infected *inos* ^*-/-*^ mice.

### T cells are required for iNOS-mediated protection during infection

**Fig 7:**
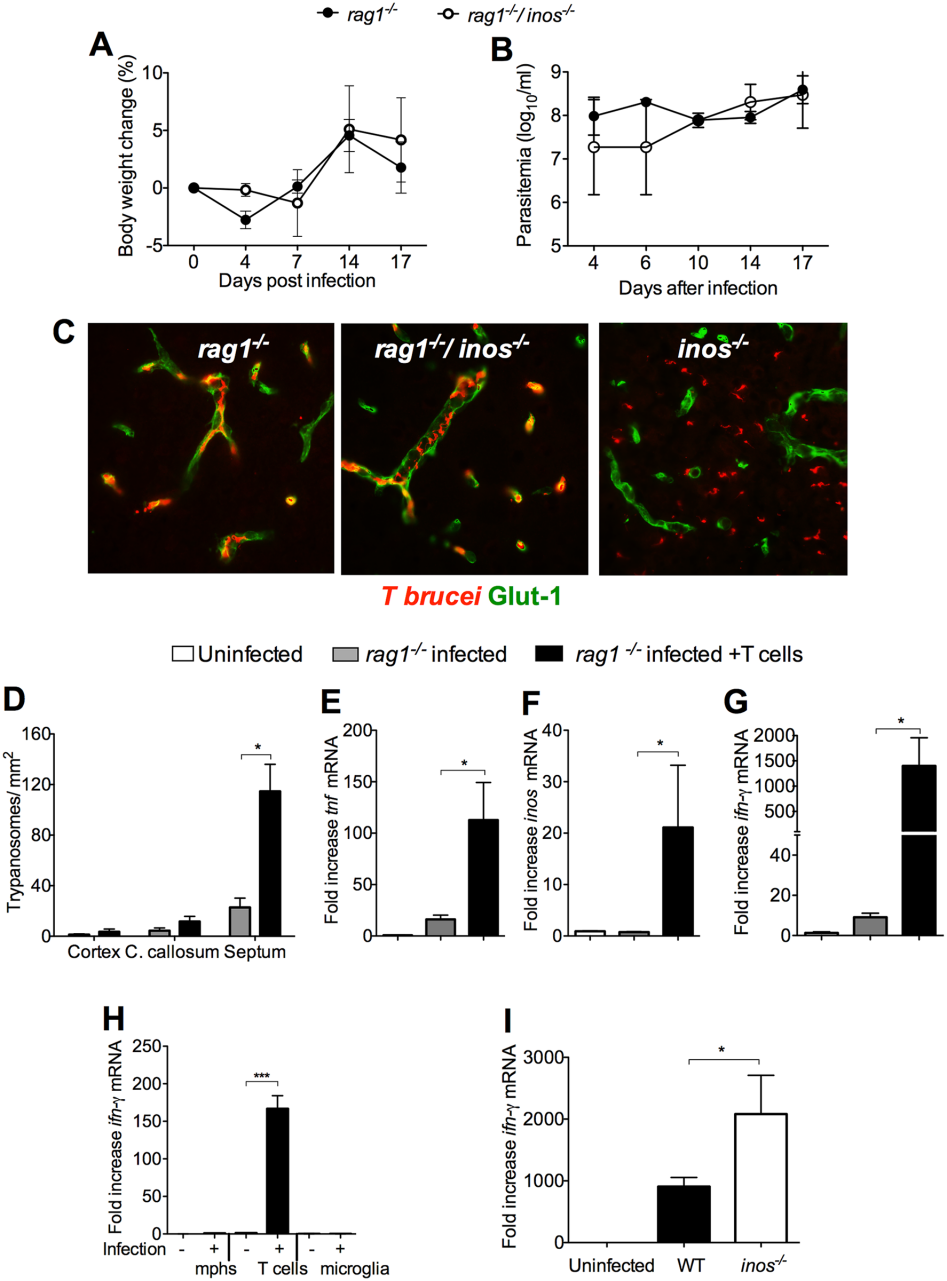
iNOS hampers T cell-mediated parasite penetration into the brain parenchyma. (A, B) Mean body weight and log_10_ parasites per ml ± SEM of *rag1* ^-/-^/ *inos* ^*-/-*^ and *rag1* ^*-/-*^ mice (n = 9–10) infected with *T*.*b*. *brucei*. (C) Representative immunofluorescence images from the septal nuclei showing *T*.*b*. *brucei* in red and cerebral endothelial cells in green of *rag1* ^*-/-*^, *rag1* ^-/-^/ *inos* ^*-/-*^ and *inos* ^*-/-*^ mice at 22 dpi. (D) Quantification of *T*.*b*. *brucei* invasion in the cerebral regions *rag1* ^*-/-*^ mice inoculated or not with 5 x10^6^ CD90^+^ T cells i.v. 7 days before infection with *T*.*b*. *brucei*. The mean number of parasites ± SEM (n = 6 per group) in T cell inoculated and non-transferred controls in one of two independent experiments is depicted. The accumulation of *tnf* (E), *inos* (F) and *ifng* (G) transcripts in infected and T cell-transferred *rag1* ^*-/-*^ mice and controls at 23 dpi. The mean fold of mRNA increase ± SEM in brains from infected mice (n ≥ 5 per group) was calculated. Differences with controls are significant (*p<0.05 Student’s t test). (H) RNA was extracted from FACS sorted from macrophage-, microglia- and T cell-enriched brain populations from *T*. *brucei*-infected and control mice as described in materials and methods. The mean fold *ifng* mRNA increase ± SEM of 4 independent pools per group is depicted. (I) The mean fold increase of *ifng* mRNA ± SEM in RNA from brains from infected WT or *inos* ^*-/-*^ and uninfected mice (n ≥ 4 per group) was measured. Differences with controls are significant (***p<0.001 Student’s t test).

Whether T cells also played a role in the iNOS-mediated inhibition of parasite penetration into the brain was then studied. B and T cell deficient *rag1* ^*-/-*^ and *rag1* ^*-/-*^/ *inos* ^*-/-*^ mice showed similar body weight loss and parasitemia levels after infection (Fig 7A and 7B). Both *rag1* ^*-/-*^ and *rag1* ^*-/-*^/ *inos* ^*-/-*^ mice showed very few parasites in the brain parenchyma, indicating that T and/ or B cells are required for the increased parasite penetration into the brain of *inos* ^*-/-*^ mice (Fig 7C). In line with this, infected *rag1* ^*-/-*^ mice transferred with T cells showed increased parasite density in the brain parenchyma (Fig 7D). *Tnf*, *inos and ifng* mRNA levels were all elevated in the brains of infected *rag1* ^*-/-*^ mice transferred with T cells compared to non-transferred infected controls (Fig 7E–7G). In agreement, *cxcl10* and *ccl2* mRNA levels were elevated in *rag1* ^*-/-*^ seeded with T cells as compared to controls (S7A and S7B Fig). Brains from either *tnfr1* ^*-/-*^ or *inos* ^*-/-*^ /*tnfr1* ^*-/-*^ mice during infection with *T*.*b*. *brucei* contained diminished levels *cxcl10* and *ccl2* mRNA as compared to those of *inos* ^*-/-*^ mice (S7C and S7D Fig). The accumulation of *cxcl10* and *ccl2* mRNA in LPS-stimulated *inos* ^*-/-*^ BMM was increased in comparison to that of WT controls (S7E and S7F Fig), suggesting an inhibitory role of iNOS-derived NO in the expression of these chemokines.

High levels of *ifng* mRNA were found in T cell-enriched populations from brains of infected mice (Fig 7H) but not in macrophages or microglia. Thus, T cells express IFN-γ in the brain and contribute to the induction of TNF and iNOS expression by brain macrophages. Of note, *ifng* mRNA expression was increased in the brains of *inos* ^*-/-*^ mice infected with *T*.*b*. *brucei* (Fig 7I). Thus iNOS-derived NO hampers a T cell-mediated cerebral vascular leakage.

In summary, both TNF and activated T cells are necessary to stimulate iNOS expression by brain macrophages, while reciprocally iNOS-derived NO dampens a TNF and T cell-mediated brain invasion of parasites and leukocytes.

## Discussion

In the present study, we describe a protective, anti-inflammatory role of NO in the pathogenesis of an experimental neuroinflammatory disease, i.e. African trypanosomiasis. In particular, iNOS-produced NO was critical for maintaining the integrity of the BBB, hampering continuous brain invasion of parasites and leukocytes. This was unexpected, since release of iNOS-derived NO into the brain often has been associated with toxicity both of the BBB and the nervous tissue.

The infection with *T*.*b*. *brucei* stimulated the presence of nitrites and nitrosylated proteins in the plasma and brains. Although previous studies have indicated that a variety of cells in the nervous system may produce iNOS, e.g. perivascular macrophages, microglia, astrocytes and even neurons [26], we found that in the brain parenchyma iNOS was most prominently expressed in perivascular macrophages. This is an ideal site for interactions with trafficking of inflammatory cells across the BBB that occurs at the level of postcapillary venules [27]. Inflammatory cells cross the BBB at this level either after a transient opening of the tight junctions between the endothelial cells or via transcytosis though the endothelium. Both mechanisms have been suggested for passage of trypanosomes [28].

*Inos* ^*-/-*^ and control mice showed similar parasitemia levels during the encephalitic stage of infection with *T*.*b*. *brucei*. A parasite-released factor that antagonize conversion of L-arginine to NO through stimulation of arginases of myeloid cells suggest the relevance of this pathway[29]. In addition, trypanothione, a parasite glutathione-like compound that confers protection against oxidative stress, may protect the parasite by sequestering NO and iron into a harmless stable complex [30]. The suggestion that NO has no major parasiticidal effect supports our **in vitro** observation that *T*.*b*. *brucei* was not more susceptible to NO donors than mammalian cells.

iNOS-derived NO has been shown to promote the blood flow and protect in a model of systemic shock [4]. Similar to our observations of a the beneficial effect of NO, various NO donors have recently been shown also to reduce neuroinflammation and increase cerebro-vascular flow [31]. Low rather than high NO levels have been shown to contribute to the genesis of experimental cerebral malaria (ECM). Exogenous GSNO protected against ECM, decreased pro-inflammatory biomarkers in the blood and vascular leakage into the brain, without affecting levels of parasitemia [32,33]. Polymorphisms in the gene encoding iNOS associated with malaria severity in human populations [34,35]. Moreover, the levels of NO in plasma correlated inversely with the incidence of severe malaria in human populations [35,36]. Accordingly, the protection against neuroinflammation observed in *T*.*b*. *brucei* infected *inos* ^*-/-*^ mice treated with GSNO therefore confirms that inos derived NO controls susceptibility to infection.

In ECM and African trypanosomiasis TNF has been shown to be crucial for parasite control [37], but has also been associated with the severity of neurological symptoms in the human disease [38]. We found that macrophages, microglia and T cells in the brain of *T*.*b*. *brucei*-infected mice produce TNF. In line with this, a population of CD11c^+^ Ly6c^+^ monocyte-derived cells was shown to be the major source of systemic TNF during *T*.*b*. *brucei* infection[39]. Our data corroborate the notion that TNF is required for iNOS expression and that TNF mediates parasite and T cell penetration into the brain [7]. Most notable, we show a negative feedback loop in which iNOS-derived NO dampens both the expression and the signaling of TNF. NO inhibition of TNF signaling has been previously shown on LPS-stimulated hepatocytes [40]. TNF-RI signaling is also required for neuroinvasion and extravasation of proteins in *inos* ^*-/-*^ mice since the outcome of infection in *inos* ^*-/-*^/ *tnfr1* ^*-/-*^ is similar to that of *tnfr1* ^*-/-*^ mice. Corroborating our observations, NOS inhibition or iNOS-deficiency has been shown to exacerbate TNF toxicity in a sepsis model by downregulating IFN-γ and/ or TNF production [4,41].

The S-nitrosylation of numerous transcription factors is crucial for the control of mammalian gene transcription[42]. The p50-p65 heterodimer is the prototypical member of the NF-κB family that mediates the expression of and responses to TNF. NO inhibits NF-κB-dependent DNA binding through S-nitrosylation of p50 and p65 that are involved in TNF gene transcription [43–45]. Our experiments indicate that NO mediates S-nitrosylation and inhibits NF-κB p65 activation in the brain of infected mice. Our experiments do not exclude the possibility that NO also inactivates other molecules regulating NF-κB activation or proteins in signalling pathways that cross talk with NF-κB and with TNF or MMP9 expression [46–48].

MMP is a family of structurally related zinc-dependent endopeptidases capable of degrading ECM and basement membrane, both in physiological and pathological events. TNF has been shown to induce MMP-9 expression, which, in turn, by cleaving a β-dystroglycan receptor that anchors astrocytic endfeet to the parenchymal basement membrane of postcapillary vessels is crucial for leukocyte infiltration into the brain [50]. We found here that expression of MMP9 in the brains from infected mice **in vivo** and in macrophage cultures **in vitro** was dependent on TNF and increased in the absence of iNOS. While *mmp9* mRNA accumulation and generic MMP activity are elevated, whether this results in higher MMP9-zymogen levels in the brain of *inos* ^*-/-*^ infected mice remains to be determined. Minocycline, with proven MMP inhibitory functions [51], hampered MMP9 but not TNF gene expression and protected both WT and *inos* ^*-/-*^ mice from leukocyte and *T*.*b*. *brucei* invasion into the brain. In line with this, in mice afflicted with EAE and treated with minocycline exhibited reduced expression and activity of MMP9 and such mice showed alleviated neuropathological changes [25]. However, other molecules targeted by minocycline may also contribute to *T*. *brucei*-induced neuroinflammation.

Finally we confirmed that T cells are required for *T*. *brucei* penetration into the brain [9]. Furthermore, iNOS is induced by T cells but also hampers T cell-mediated responses indicating that reciprocal regulatory mechanisms exists between NO and T cells [52]. IFN-γ is required for brain invasion during *T*.*b*. *brucei* infection [9]. Brains from infected *inos* ^*-/-*^ mice contained higher levels of IFN-γ, suggesting that iNOS inhibition of IFN-γ secretion accounts for the obstruction of T cell and parasite invasion into the brain. T cells and TNF also regulated *cxcl10* and *ccl2* gene expression in the brain of *T*.*b*. *brucei-*infected mice. Transcripts of these chemokines were elevated in the brain of infected *inos* ^*-/-*^ mice. Increased levels of these chemokines might also contribute to the enhanced inflammation in infected *inos* ^*-/-*^ mice.

**Fig 8:**
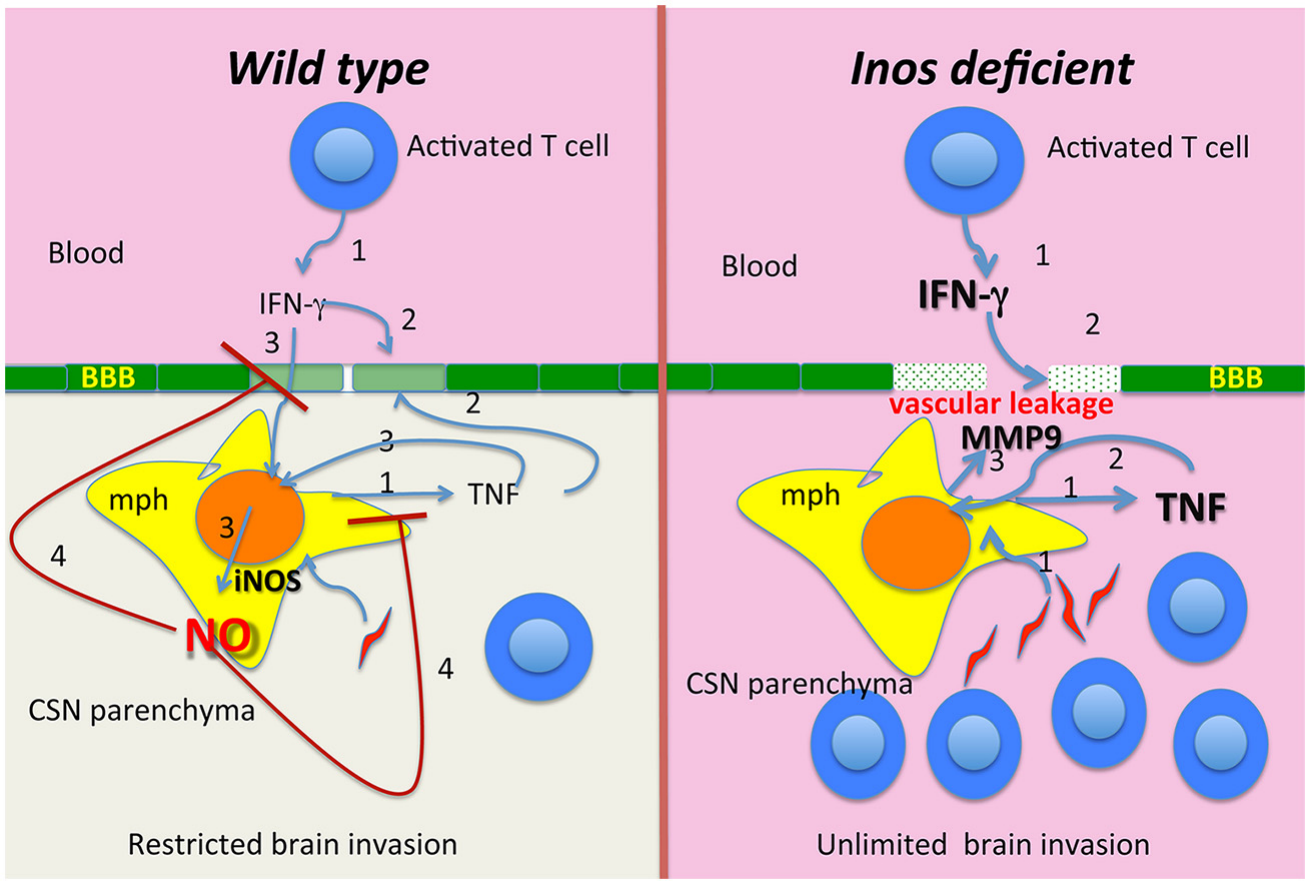
Graphical summary. In wild type mice, *T*.*b*. *brucei* infection stimulates the expression of TNF and IFN-γ by macrophages and T cells respectively (1). T cells and TNF are required for brain invasion (2), but are also non-redundant stimulators of iNOS expression in perivascular macrophages (3). iNOS-derived NO harnessed the expression of and the response to TNF, and T cell mediated brain invasion by parasites and leukocytes (4). Nitrosylation of intracellular signalling molecules such as NF-κΒ p65 might lead to diminished TNF release and signalling (4). In the absence of iNOS (right panel), TNF levels increased resulting in an amplified MMP9 expression (3) that mediates the BBB breakdown followed by vascular leakage and an uncontrolled penetration of T cells and parasites into the brain.

In conclusion, our findings show that iNOS harnessed a T cell- and TNF-mediated neuroinflammation. NO nitrosylated intracellular signalling molecules such as NF-κB p65 leading to a diminished TNF release and signalling. TNF levels augmented in the absence of iNOS resulting in increased expression of MMP9, which mediated enhanced invasion of trypanosomes and leukocytes across the BBB (Fig 8). Thus, iNOS derived NO prevents an un-limited crossing of inflammatory cells and the leakage of serum components into the brain parenchyma that could cause nervous system dysfunctions and degeneration.

## Materials and Methods

### Ethics statement

The animals were housed and handled at the Dept of Microbiology, Tumor and Cell Biology and the Astrid Fagreus Laboratory, Karolinska Institutet, Stockholm, according to directives and guidelines of the Swedish Board of Agriculture, the Swedish Animal Protection Agency, and the Karolinska Institute (djurskyddslagen 1988:534; djurskyddsförordningen 1988:539; djurskyddsmyndigheten DFS 2004:4). The study was performed under approval of the Stockholm North Ethical Committee on Animal Experiments permits number 187/ 12 and 35/15. Animals were housed under specific pathogen-free conditions.

### Mice and parasites

*Inos* ^-/-^, *tnfr1* ^*-/-*^ and *rag1* ^-/-^ mice were generated by homologous recombination in embryonic stem cells. *Rag1* ^*-/-*^ */inos* ^*-/-*^ *and tnfr1* ^*-/-*^ */inos* ^*-/-*^ mice were generated by cross breeding of *inos* ^*-/-*^ and *rag-1* ^*-/-*^ or *inos* ^*-/-*^ and *tnfr1* ^*-/-*^ mice. All strains were backcrossed on a C57BL/6 background, and C57BL/6 mice were used as wild type (WT) controls.

Mice (6–8 weeks old) were infected by intraperitoneal (i.p.) injection with 2 x10^3^ parasites of a pleomorphic stabilate of *T*.*b*. *brucei*, AnTat 1.1E (obtained from ITG, Antwerp, Belgium). Parasitemia levels were determined by microscopy counting of tail vein blood and body weight monitored during the course of the infection.

The NO donor S-nitrosoglutathione (GSNO), a physiological metabolite of reduced glutathione (GSH) and NO, was synthesized as described [53]. Mice were daily treated i.p. with 3.5 mg GSNO starting on day 5 after *T*. *brucei* infection.

When indicated, mice were treated i.p. daily with minocycline (Sigma, St Louis, MO) or its vehicle (PBS), commencing on the day of *T*. *b*. *brucei* inoculation. The infected mice received 50 mg of the minocycline/ kg twice a day for the first 2 days and once daily for the next 5 days, followed by 25 mg/kg for the subsequent days until the animals were sacrificed.

### Generation of mouse bone marrow-derived macrophages (BMM)

Bone marrow was extracted from tibia and femur of mice and resuspended in Dulbecco’s modified Eagle’s medium (DMEM) containing glucose and supplemented with 2 mM L-glutamine, 10% FCS, 10 mM Hepes, 100 μg/ml streptomycin, 100 U/ml penicillin (all from Sigma), and 30% L929 cell-conditioned medium (source of macrophage-colony stimulating factor). Bone marrow cells were passed through a 70 μm cell strainer, plated and incubated for 6 days at 37°C, 5% CO_2_. Bone marrow-derived macrophage (BMM) cultures were then washed vigorously to remove non-adherent cells, trypsinized, counted and cultured for one day at 37°C. We have previously shown by immunofluorescence labelling that these BMM are F4/80^+^, CD14^+^ and Mac-3^+^ [54]. BMM were subsequently stimulated with LPS (1 μg/ml) or heat killed *T*.*b*. *brucei* trypomastigotes (MOI 5:1). When indicated, BMM were treated with GSNO (200 μM) and L-cysteine (400 μM) overnight, prior to addition of LPS.

### Immunohistochemistry

To examine passage of trypanosomes across the BBB, 14 μm vertical sections of fresh frozen brains at levels including the septal nuclei were cut, mounted and fixed in 4% formalin with 0.17% picric acid in PBS followed by acetone. The sections were double labeled by incubation with either antibodies recognizing the parasite, cerebral endothelial and inflammatory cells or plasma proteins (S2 Table), followed by fluorochrome labelled secondary antibodies rhodamine red donkey anti-rat IgG, anti-rabbit IgG, or anti mouse IgG and Alexa Fluor488 donkey anti-goat IgG *(*all from Jackson Immunoresearch, West Grove, PA). Sections were examined in a Leica DMRE fluorescence microscope.

### Real Time PCR

Total RNA was extracted from brain samples or culture cells using Trizol and cDNA was obtained by reverse transcription. Transcripts were quantified by real time PCR as previously described [55]. *Hprt* was used as a control gene to calculate the ΔCt values for individual brain samples. The relative amount of cytokine/ *hprt* transcripts was calculated using the 2^-(ΔΔCt)^ method. These values were then used to calculate the relative expression of cytokine mRNA in uninfected and infected cells and tissues. The sequences of primers used are shown in S3 Table.

### Flow cytometry and cell sorting

Brains were removed, minced and incubated at 37°C, 5% CO2 with an enzymatic solution containing 20 units/ml papain. After 90 min, the brain suspensions were washed in HBSS 20%FBS, incubated with DNase I (0.5mg/ml, Roche) and filtered through a 40-μm nylon cell strainers. Myelin removal beads (Miltenyi Biotech, Bergisch Gladbach, Germany) were used to remove myelin debris from the dissociated tissue. Cells were stained for CD3, Ly6C, Ly6G, CD11b and CD45 using fluorochrome labelled antibodies and fixed before acquisition.

Brain CD45^high^CD11b^-^ enriched in T cells, CD45^high^CD11b^+^ macrophage-enriched cells and CD45^dim^CD11b^+^ microglia [56] were separated by FACS sorting (MoFlo XDP, Beckman Coulter, Brea, CA). Sorted cells (ca 2x10^5^) were collected in RNA lysis buffer and RNA was extracted using RNAeasy micro kit (Qiagen, Limburg, Netherlands). Microglia from uninfected mice served as control. Since T cells are not present in uninfected animals, CD90+ T cells were obtained from spleens from uninfected mice and used as controls. BMM were used as negative controls for brain macrophages given the very low amount of these populations in the brain of uninfected animals. Real time PCR analysis was performed as described above.

For identification of iNOS-producing cells, brain cell suspensions were fixed, permeabilized using leukocyte permeabilization reagent (IntraPrep, Immunotech, Marseille, France) and stained with anti-iNOS-specific antibodies (BD). Data were acquired in CyAn ADP flow cytometer (Beckman Coulter) and analyzed using FlowJo software (Tree star Inc., Ashland, OR).

### Assessment of blood-brain barrier permeability disturbances in vivo

Permeability of the BBB was visualized using Evans blue (EB), which binds serum albumin, as a tracer [57]. 300 μl of 3% EB 5% BSA in PBS solution were administered i.p.. EB binds to albumin in blood and can be used as a quantitative marker for tracing this protein. Four hours post-injection the mice were sacrificed and the brains were removed, fixed in 4% paraformaldehyde and then incubated in 10% sucrose, 0.02% bacitracin, 0.01% sodium azide, 0.1M phosphate buffer for 48–72 hrs. Cryostat sections (12 μm) were cut and EB was visualized by its emission of red fluorescence under microscopy.

To quantify extravascular accumulations, of EB, or immunolabelled IgG or fibrin the immunofluorescence was subjected to threshold processing and measured using the “Image J” integrated density analysis tool. For this, photomicrographs (100 X) from at least 4 or more mice per group and 3 micrographs per individual were analysed.

### Nitrite/ nitrate determinations

To analyze the concentration of the stable oxidation products of NO in the supernatants and the plasma samples, the total concentration of nitrate and nitrite was calculated as previously described [58]. Briefly, nitrate was reduced by adding 10 μl of NADPH 10 μM and 40 μl of nitrate reductase (10 U/ml in PBS) to 50 μl of the plasma for 45 min at room temperature. The Griess reaction was performed by adding 100 μl of 1% (w/v) sulfanilamide in 5% phosphoric acid followed by 100 μl of 0,1% (w/v) N-(1-naphtyl) ethylenediamine HCl to 50 μl of samples. After incubation for 10 min RT the absorbance was read at 540 nm.

### Fluorescent DAN assay

The 2,3-diaminonapthalene (DAN) assay was used to measure S-nitrosothiols in serum. In brief, 100 μM DAN and 300 μM HgCl2 were added to serum samples. After incubating at room temperature for 30 min, NaOH was added to terminate the reaction. The fluorescence of naphthotrazole, the reaction product, was measured at Ex/Em = 375/450 nm.

### MMP activity assay

The activity of matrix metalloproteinases (MMP) was measured with a fluorometric assay kits (SensoLyte 520 Generic MMP, AnaSpec, Fremont, CA). Supernatants from LPS*-*stimulated BMM (untreated and minocycline-treated) were activated with APMA during 2hs at 37°C and then mixed with 5-FAM/QXL520 FRET peptide substrate. After 1 h incubation, fluorescence was measured at Ex/Em = 490/520 nm. The relative fluorescence unit (RFU) was calculated by subtract the fluorescence background (substrate) to the samples wells.

### Biotin switch assay

The biotin switch technique was employed to detect total cellular nitrosylation level in brains or macrophage cultures as described [59]. Brains cell suspensions were prepared after discarding the myelin containing populations with a myelin removal column (Myltenyii) as described above. Cell lysates were then prepared by adding urea lysis buffer (8 M urea, 50 mM Tris pH 8.0, 1 mM EDTA) and 20 mM blocking reagent methyl methanethiosulfonate (MMTS). The lysates were incubated at 50°C for 30 min with frequent vortexing and centrifuged at 17,000 g for 10 min. Proteins in the supernatant were precipitated with cold acetone in order to remove free MMTS. The pellet was washed 3 times in cold acetone and resuspended in urea lysis buffer with 20 mM sodium ascorbate and 0.5 mM N-6-(biotinamido) hexyl-3'-(2'-pyridyldithio) propionamide (Biotin-HPDP, Thermo Scientific Pierce, Waltham, MA). The reaction was done at room temperature for 1 hr. Cold acetone was then added to remove excessive biotin-HPDP and the resulting pellet was dissolved into 2 M urea 50 mM Tris-EDTA (pH 8.0). Protein concentration was determined by BCA protein assay. Protein samples (10 μg) were loaded on a SDS-PAGE, blotted and biotinylated proteins were detected by streptavidin-HRP (Cayman Chemical, Ann Arbor, MI). To detect NF-κB p65 nitrosylation, biotinylated proteins were immuno-precipitated by high capacity neutravidin agarose resin (Thermo Scientific Pierce) at 4°C overnight. Proteins were eluted with 20 mM HEPES, pH 7.7, 100 mM NaCl, 1 mM EDTA, 100 mM DTT. Eluted proteins were loaded on a SDS-PAGE for detection of p65 by immunoblotting (NF-κB p65 antibody (sc-372, Santa Cruz Biotechnology, Dallas, TX).

In order to control the specificity of the reaction, controls in which 1) MMTS and ascorbate were added together leading to a blocking of all SH and SNO groups in the proteins, and 2) the reactions in presence of the DTT were performed.

### Western blot

Brains from infected and control mice were lysed and separated on 10% separating/ 5% stacking SDS-polyacrylamide gels. Samples were then transferred onto nitrocellulose membranes (BioRad, Hercules, CA) by electroblotting at 100 V, 250 mA for 80 min. Immunostaining was performed using polyclonal goat anti mouse IgG-HRP or monoclonal anti-GAPDH-HRP (Sigma). Membranes were then washed and developed using ECL-Plus (Amersham Biosciences, Buckinghamshire, UK) and photographed using a Fuji intelligent dark box II digital camera.

### Statistical analysis

The statistical tests were performed using the Prism software (GraphPad, La Jolla, CA).

## Supporting Information

S1 Fig The penetration of parasites in the brain of *inos* ^*-/-*^ does not occur at early time points of infection. (A) The cumulative mortality of WT and *inos* ^-/-^ mice infected with 2000 *T*. *b*. *brucei* parasites is depicted. Survival curves are different (Log-rank test p<0.005). (B) Representative immunofluorescence images showing *T*.*b*. *brucei* (red) and cerebral endothelial cells (green) in the cortex of *inos* ^*-/-*^ mice at the indicated days after infection.
(TIF)

S2 Fig Increased astrogliosis and presence of neuronal degeneration in the brain of *inos* ^*-/-*^ *T*.*b*. *brucei*-infected mice. (A) Immunolabelling of occludin, ZO-1 and claudin-5 (red) in cerebral microvessels (green) of WT and *inos* ^-/-^ mice 23 dpi with *T*.*b*. *brucei*. (B, C) The mean fold *occludin* (B) and *claudin-5* (C) mRNA increase ± SEM in brains from control and infected mice (n ≥ 4 per group) was calculated. (D) Immunolabelling of GFAP showing increased reactive astrogliosis in the brain of a *T*.*b*. *brucei* infected *inos* ^*-/-*^ mouse as compared to a WT mouse 23 dpi. (E) *Iba1* mRNA levels in brains of WT and *inos* ^*-/-*^ *T*.*b*. *brucei-*infected mice. (F) Representative micrographs indicating ß-APP expression in the brain parenchyma of uninfected, WT and *inos* ^*-/-*^ *T*. *brucei*-infected mice. A brain section from an Influenza A WSN/33 infected mouse was used as a positive control. (G) Immunolabelling showing similar NG2 staining labelling pericytes (arrows) in the periphery of brain vessels of a *T*.*b*. *brucei* infected WT and *inos* ^*-/-*^ and an uninfected WT animal.
(TIF)

S3 Fig CD45^high^CD11b^-^ cells are enriched in T cells, while macrophages are enriched in the CD45^high^CD11b^+^ cells. (A) The frequency of CD45^high^ CD11b^+^ (R1), CD45^dim^ CD11b^+^ (R2) and CD45^high^ CD11b^-^ (R3) within a brain non-myelin cell suspension of WT mice was determined by FACS analysis at 30 dpi. (B-D) CD3^+^ (T cells), Ly6C^+^ (monocytes, macrophages, granulocytes and also effector T cells [60]) and Ly6G^+^ (granulocytes) FACS plots in R1-3 gated subpopulations are shown.
(TIF)

S4 Fig Cytokine, chemokine and adhesion molecule transcript levels in the brain of WT and inos^-/-^ *T*.*b*. *brucei* infected mice. (A, C, E-K) The total RNA was extracted from brains of WT and *inos* ^*-/-*^ *T*.*b*. *brucei-*infected mice sacrificed 23 dpi or uninfected controls. (B, D) In other sets of experiments RNA was isolated from infected *inos* ^*-/-*^ mice treated daily with 3.5 mg GSNO starting 5 dpi. The accumulation of *vcam-1* (A, B), *icam-1* (C, D), e-*selectin* (E), *il-1b* (F), *il-6* (G), *il-17a* (H), cxcl9 (I), *cxcl0* (J), *ccl2* (K) or *hprt* transcripts was measured by real time PCR. The mean fold of either adhesion molecule or cytokine mRNA increase ± SEM in brains from infected mice (n ≥ 4 per group) was calculated. Differences with WT infected controls are significant (*p<0.05 Student’s t test).
(TIF)

S5 Fig Neither iNOS-derived NO nor addition of GSNO regulate phosphorylation of MAPK-p38. The levels of total, phosphorylated MAPK-p38 and GAPDH were analysed by western blot in lysates from WT or *inos* ^*-/-*^ BMM at different time points after stimulation with 1 μg/ml LPS, in presence or absence of 200 μM GSNO.
(TIF)

S6 Fig *Mmp9* transcript levels are increased in the macrophage-enriched brain subpopulations after infection with *T*.*b*. *brucei*. RNA was extracted from FACS sorted from (A) macrophage-, (B) microglia- and (C) T cell-enriched brain populations from *T*. *brucei*-infected and control mice as described in material and methods. The mean fold *mmp9* mRNA increase ± SEM of 4 independent pools per group are depicted. Differences with controls are significant (***p<0.001 Student’s *t* test).
(TIF)

S7 Fig *Cxcl10* and *cxcl2* mRNA levels are increased in the brains of *T*. *brucei*–infected *inos* ^*-/-*^ mice, and in LPS-stimulated *inos* ^*-/-*^ BMM. The accumulation of *cxcl10* (A) and *ccl2* (B) transcripts in T cell-transferred or control *rag1* ^*-/-*^ mice was measured at 23 dpi. The mean fold of mRNA increase ± SEM in brains from infected mice (n ≥ 5 per group) was calculated. The accumulation of *cxcl10* (C) and *ccl2* (D) mRNA in brains from *inos* ^*-/-*^, *inos* ^*-/-*^ /*tnfr1* ^*-/-*^ and *tnfr1* ^*-/-*^ mice (n≥6) was measured 22 days after infection with *T*. *brucei*. The levels of *cxcl10* (E) and *ccl2* (F) mRNA was measured in total RNA extracted from *inos* ^*-/-*^ or WT BMM independent cultures (n = 3) 24 after LPS stimulation and repeated in two independent experiments. Differences with controls are significant (*p<0.05, **p<0.01 Student’s t test).
(TIF)

S1 Table Toxicity of NO donors SNAP and GSNO on *T*. *brucei* and mammalian cell lines. Parasites and mammalian cell lines were incubated with serial dilutions of SNAP (S-nitroso-N acetylpenicillamine) or GSNO (S-nitrosoglutathione)). The IC_50_ was determined 72h after incubation with the compounds.
(DOCX)

S2 Table List of specific antibodies used. (DOCX)

S3 Table List of primer sequences and gene ID numbers. (DOCX)

S1 Text Supplementary experimental procedures. (DOCX)
