# Supplementary figures and images for: Nitric Oxide Protects against Infection-Induced Neuroinflammation by Preserving the Stability of the Blood-Brain Barrier
Source: PLoS Pathog. 2016 Feb 25;12(2):e1005442. doi: 10.1371/journal.ppat.1005442 (PMC4767601; doi:10.1371/journal.ppat.1005442)

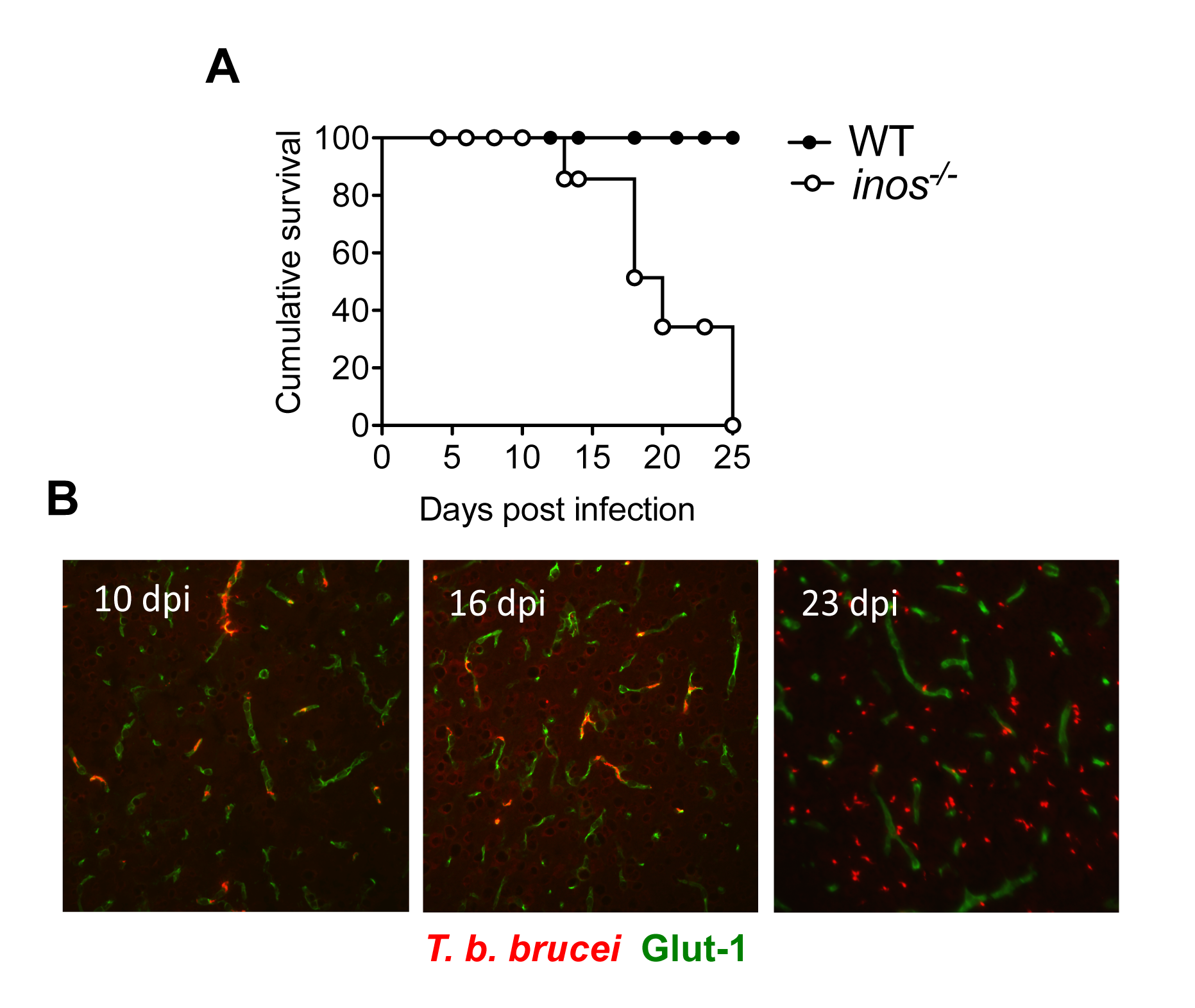

Supplement: S1 Fig — (A) The cumulative mortality of WT and inos -/- mice infected with 2000 T. b. brucei parasites is depicted. Survival curves are different (Log-rank test p<0.005). (B) Representative immunofluorescence images showing T.b. brucei (red) and cerebral endothelial cells (green) in the cortex of inos -/- mice at the indicated days after infection. (TIF) [file ppat.1005442.s001.tif]

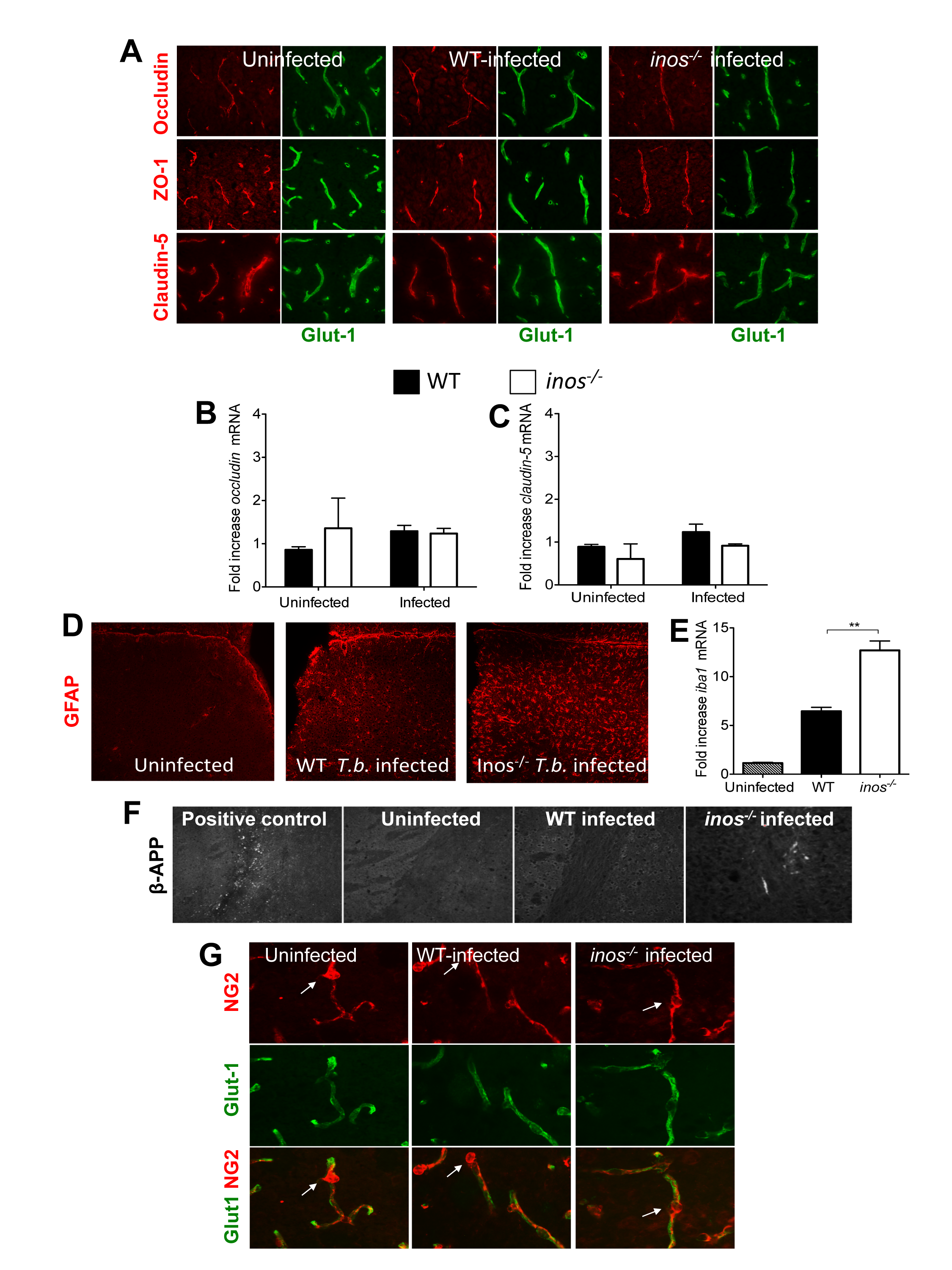

Supplement: S2 Fig — (A) Immunolabelling of occludin, ZO-1 and claudin-5 (red) in cerebral microvessels (green) of WT and inos -/- mice 23 dpi with T.b. brucei. (B, C) The mean fold occludin (B) and claudin-5 (C) mRNA increase ± SEM in brains from control and infected mice (n ≥ 4 per group) was calculated. (D) Immunolabelling of GFAP showing increased reactive astrogliosis in the brain of a T.b. brucei infected inos -/- mouse as compared to a WT mouse 23 dpi. (E) Iba1 mRNA levels in brains of WT and inos -/- T.b. brucei-infected mice. (F) Representative micrographs indicating ß-APP expression in the brain parenchyma of uninfected, WT and inos -/- T. brucei-infected mice. A brain section from an Influenza A WSN/33 infected mouse was used as a positive control. (G) Immunolabelling showing similar NG2 staining labelling pericytes (arrows) in the periphery of brain vessels of a T.b. brucei infected WT and inos -/- and an uninfected WT animal. (TIF) [file ppat.1005442.s002.tif]

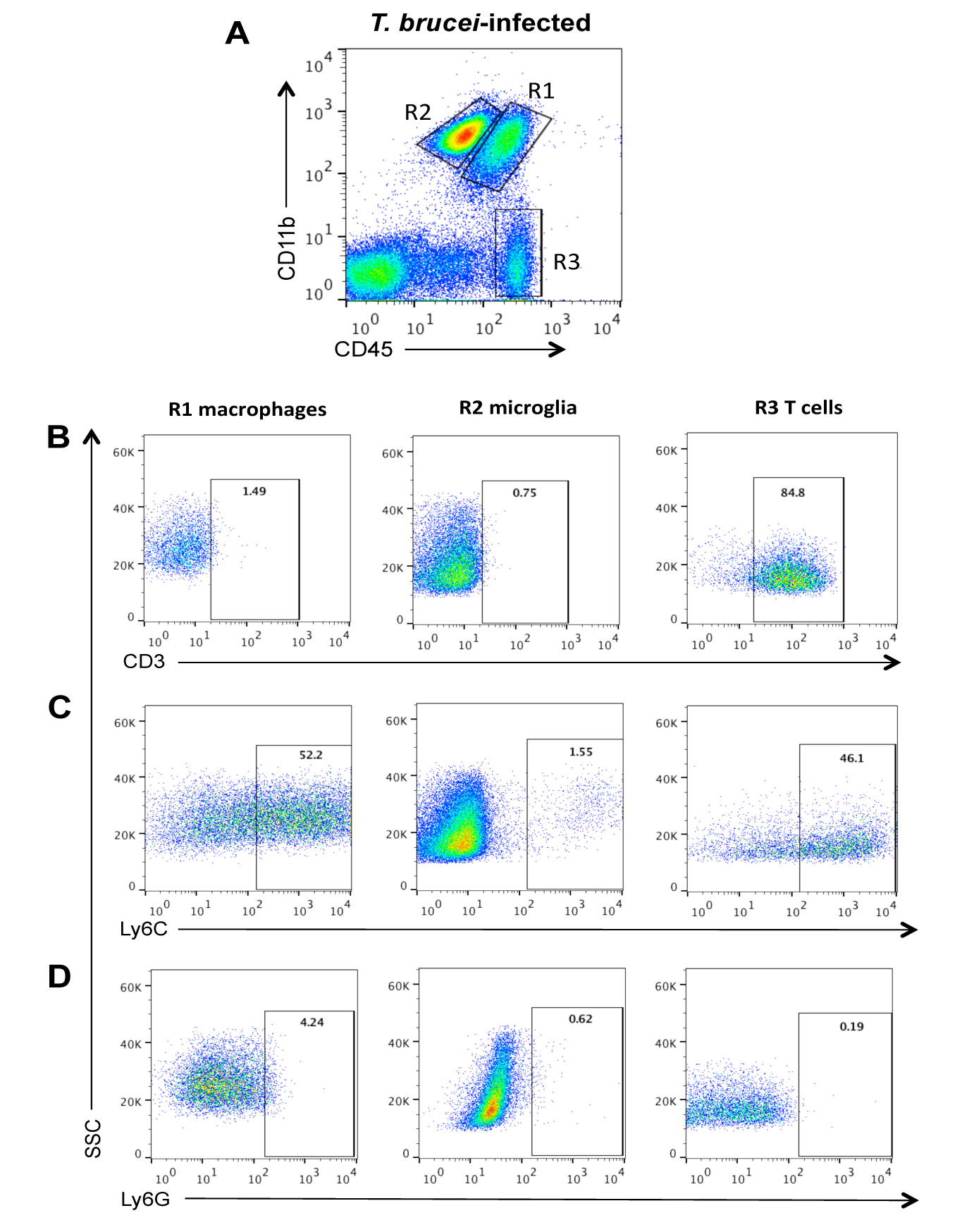

Supplement: S3 Fig — (A) The frequency of CD45high CD11b+ (R1), CD45dim CD11b+ (R2) and CD45high CD11b- (R3) within a brain non-myelin cell suspension of WT mice was determined by FACS analysis at 30 dpi. (B-D) CD3+ (T cells), Ly6C+ (monocytes, macrophages, granulocytes and also effector T cells [60]) and Ly6G+ (granulocytes) FACS plots in R1-3 gated subpopulations are shown. (TIF) [file ppat.1005442.s003.tif]

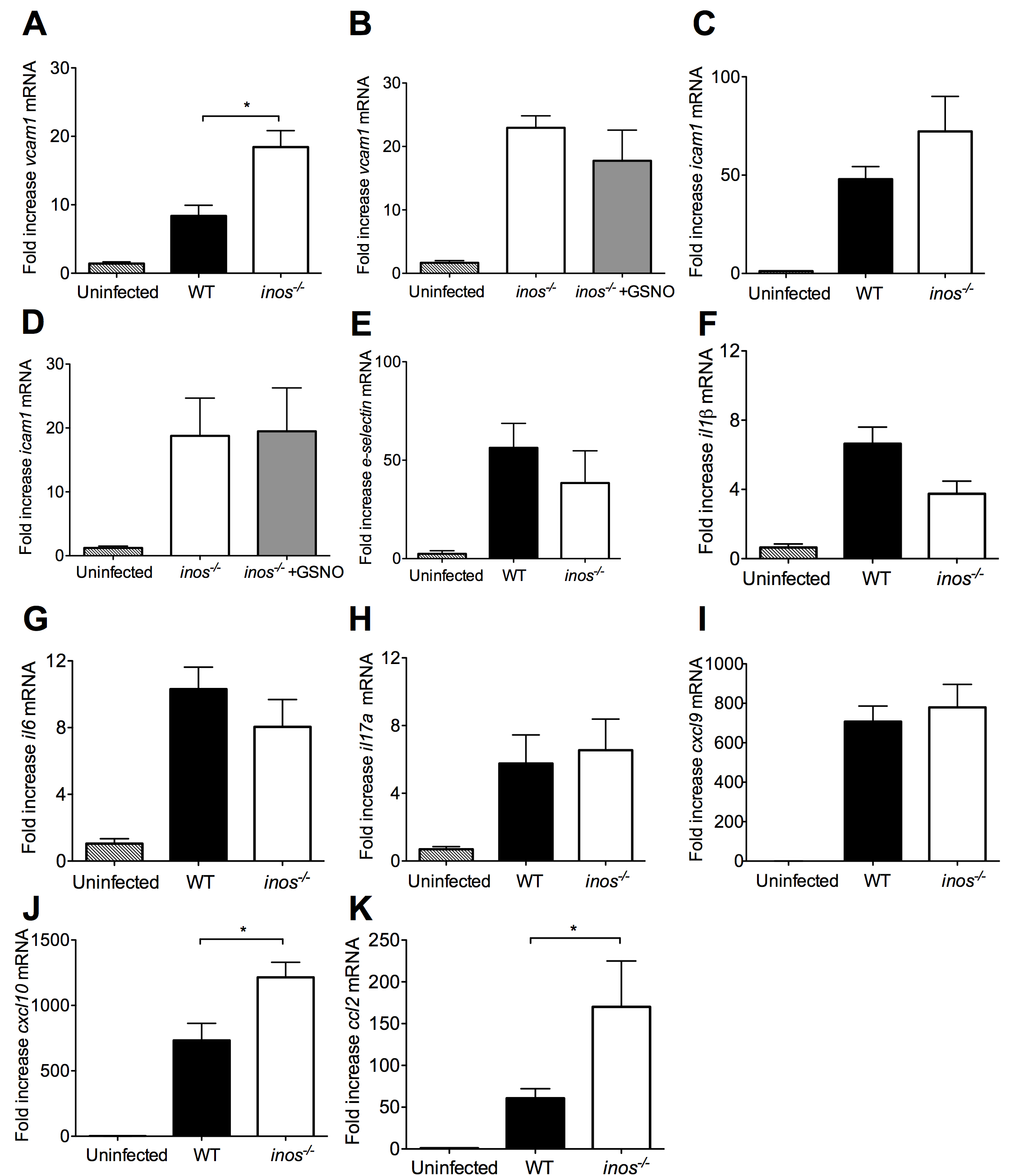

Supplement: S4 Fig — (A, C, E-K) The total RNA was extracted from brains of WT and inos -/- T.b. brucei-infected mice sacrificed 23 dpi or uninfected controls. (B, D) In other sets of experiments RNA was isolated from infected inos -/- mice treated daily with 3.5 mg GSNO starting 5 dpi. The accumulation of vcam-1 (A, B), icam-1 (C, D), e-selectin (E), il-1b (F), il-6 (G), il-17a (H), cxcl9 (I), cxcl0 (J), ccl2 (K) or hprt transcripts was measured by real time PCR. The mean fold of either adhesion molecule or cytokine mRNA increase ± SEM in brains from infected mice (n ≥ 4 per group) was calculated. Differences with WT infected controls are significant (*p<0.05 Student’s t test). (TIF) [file ppat.1005442.s004.tif]

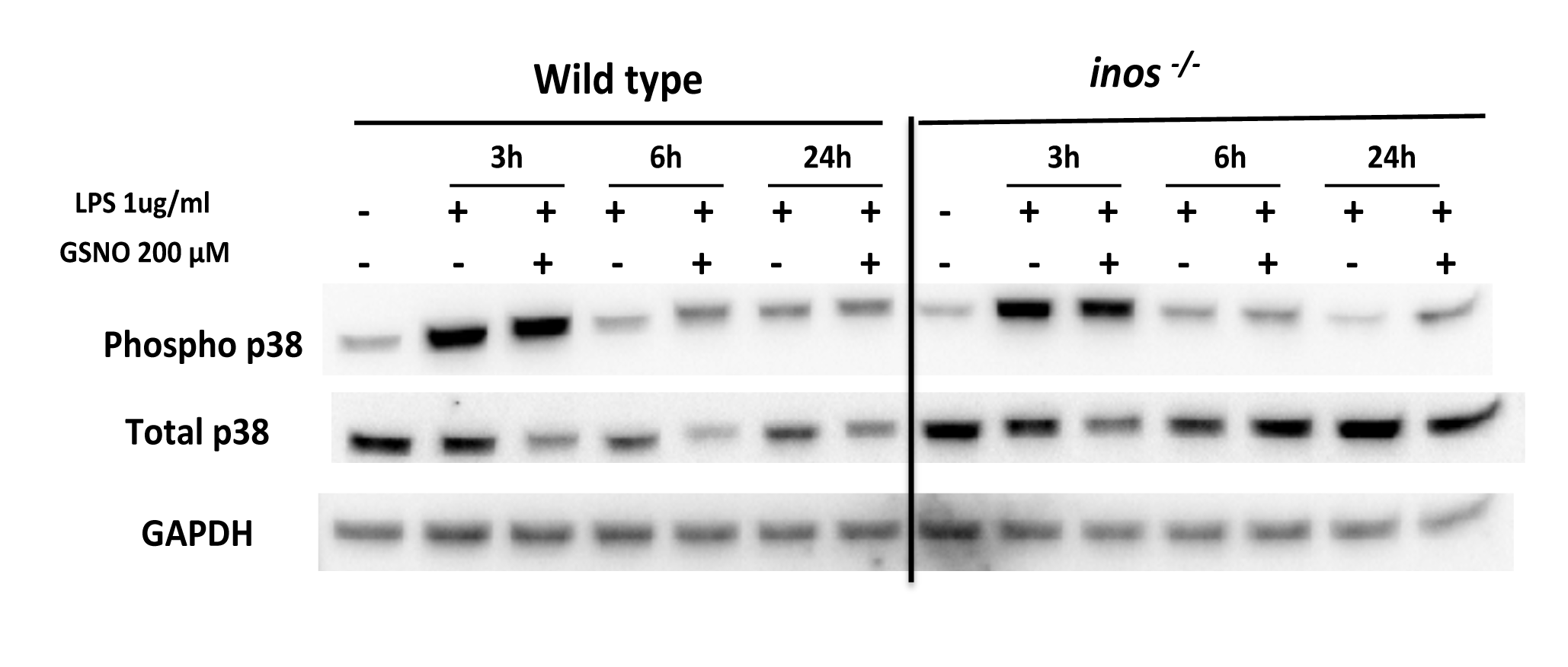

Supplement: S5 Fig — The levels of total, phosphorylated MAPK-p38 and GAPDH were analysed by western blot in lysates from WT or inos -/- BMM at different time points after stimulation with 1 μg/ml LPS, in presence or absence of 200 μM GSNO. (TIF) [file ppat.1005442.s005.tif]

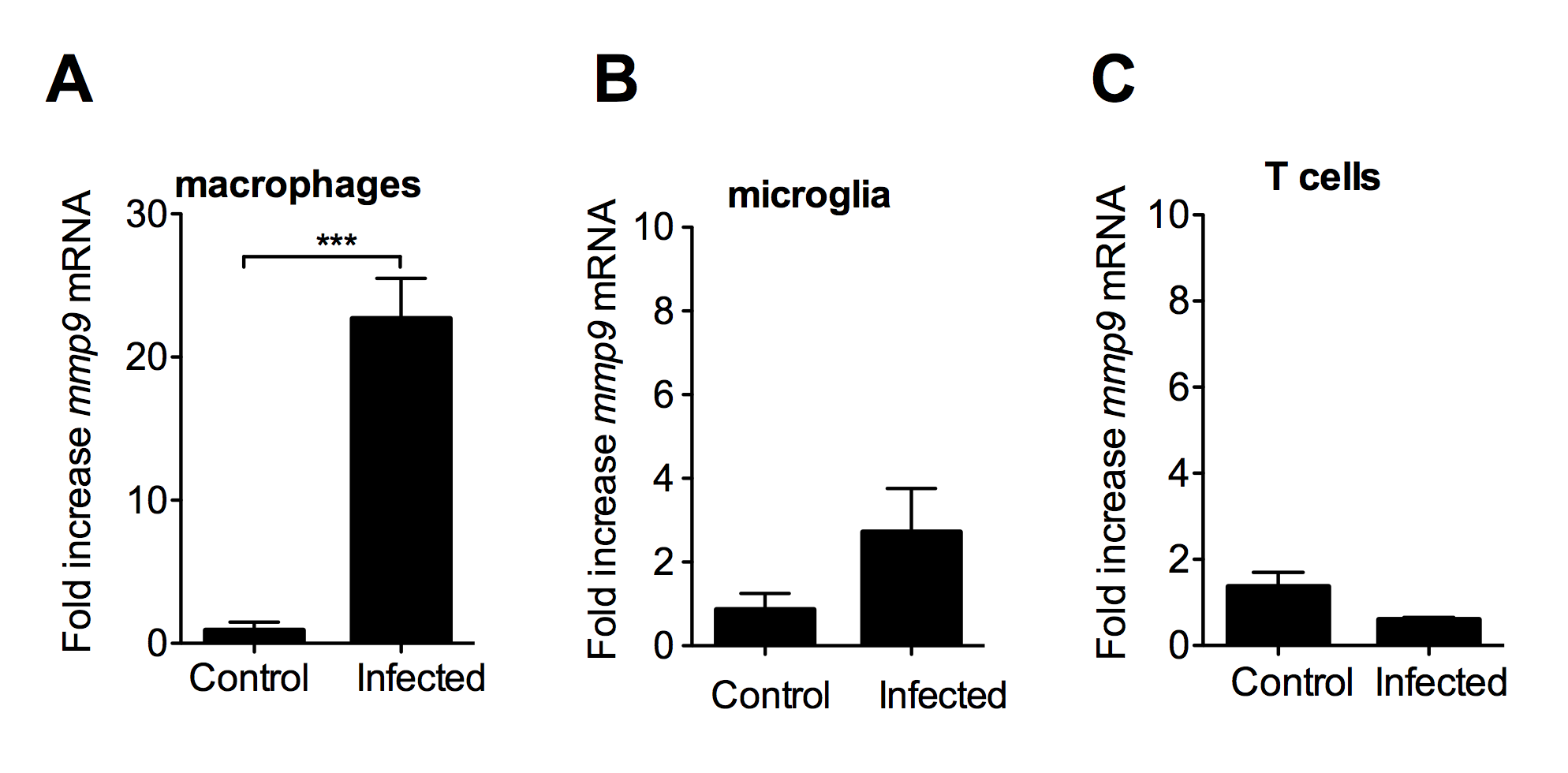

Supplement: S6 Fig — RNA was extracted from FACS sorted from (A) macrophage-, (B) microglia- and (C) T cell-enriched brain populations from T. brucei-infected and control mice as described in material and methods. The mean fold mmp9 mRNA increase ± SEM of 4 independent pools per group are depicted. Differences with controls are significant (***p<0.001 Student’s t test). (TIF) [file ppat.1005442.s006.tif]

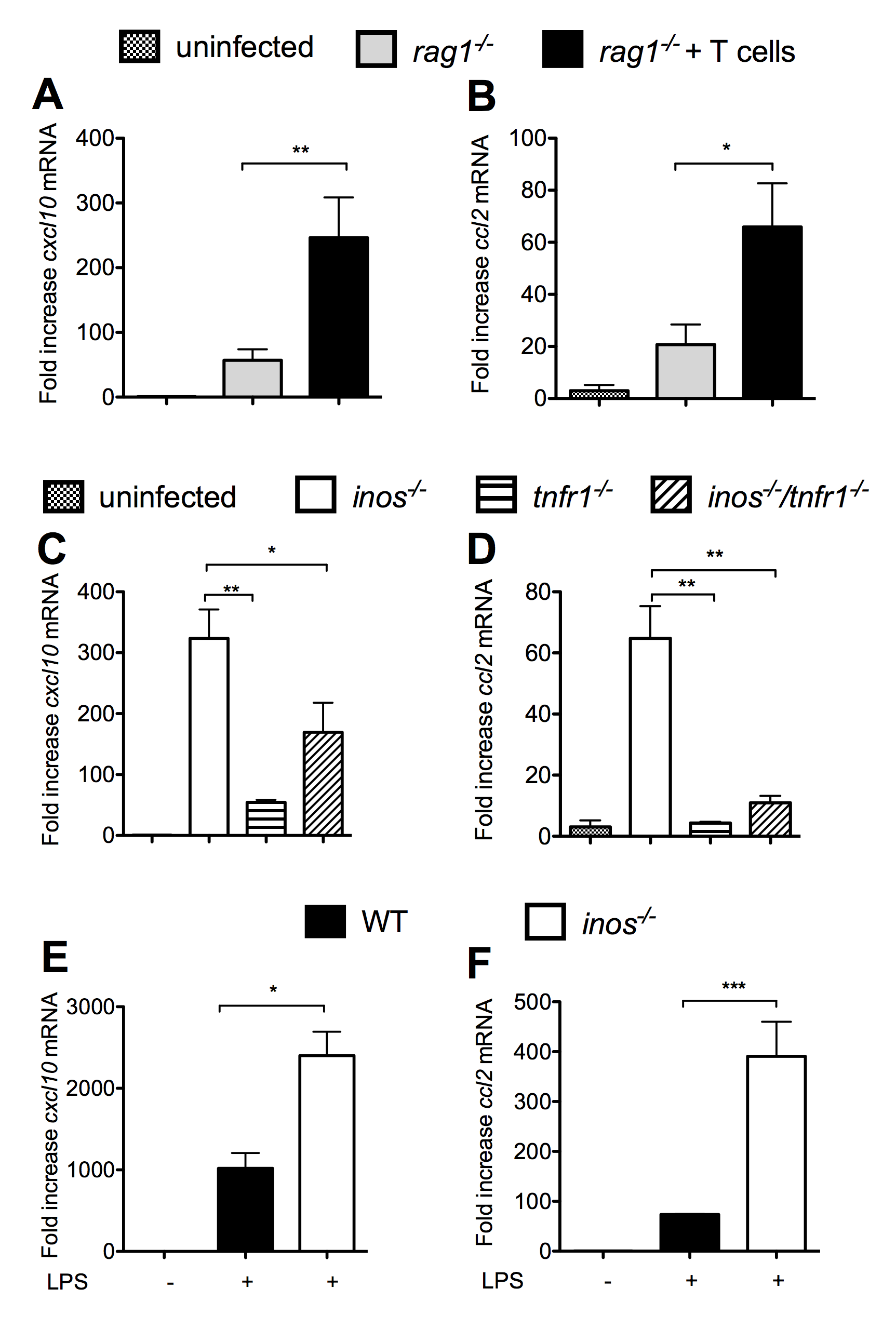

Supplement: S7 Fig — The accumulation of cxcl10 (A) and ccl2 (B) transcripts in T cell-transferred or control rag1 -/- mice was measured at 23 dpi. The mean fold of mRNA increase ± SEM in brains from infected mice (n ≥ 5 per group) was calculated. The accumulation of cxcl10 (C) and ccl2 (D) mRNA in brains from inos -/-, inos -/- /tnfr1 -/- and tnfr1 -/- mice (n≥6) was measured 22 days after infection with T. brucei. The levels of cxcl10 (E) and ccl2 (F) mRNA was measured in total RNA extracted from inos -/- or WT BMM independent cultures (n = 3) 24 after LPS stimulation and repeated in two independent experiments. Differences with controls are significant (*p<0.05, **p<0.01 Student’s t test). (TIF) [file ppat.1005442.s007.tif]
