# Supplementary material for: Nitric Oxide Protects against Infection-Induced Neuroinflammation by Preserving the Stability of the Blood-Brain Barrier
Source: PLoS Pathog. 2016 Feb 25;12(2):e1005442. doi: 10.1371/journal.ppat.1005442 (PMC4767601; doi:10.1371/journal.ppat.1005442)
Supplement: S1 Table — Parasites and mammalian cell lines were incubated with serial dilutions of SNAP (S-nitroso-N acetylpenicillamine) or GSNO (S-nitrosoglutathione)). The IC50 was determined 72h after incubation with the compounds. (DOCX) [file ppat.1005442.s008.docx]

|  | IC_50_ (µM) | |
| --- | --- | --- |
|  | SNAP | GSNO |
| *T.b. brucei* | 79.6 | 534 |
| Raw | 184 | 558 |
| Raji | 151 | 115 |
| MOLT 4 | 43 | 580 |
| TM29 | 990 | 2000 |
| HL-60 | 86 | 121 |
| GT-1 | 117 | 990 |
