# Supplementary material for: Nitric Oxide Protects against Infection-Induced Neuroinflammation by Preserving the Stability of the Blood-Brain Barrier
Source: PLoS Pathog. 2016 Feb 25;12(2):e1005442. doi: 10.1371/journal.ppat.1005442 (PMC4767601; doi:10.1371/journal.ppat.1005442)
Supplement: S2 Table — (DOCX) [file ppat.1005442.s009.docx]

| **Antibodies** | **Species** | **Target** | **Source** |
| --- | --- | --- | --- |
| An Tat 1.1 VSG | Rabbit | *T. brucei* | ITG, Antwerp |
| Anti-CD4 | Rat | CD4 | BD Biosciences, Franklin Lakes, NJ |
| Anti-CD8 | Rat | CD8 | BD |
| Anti-CD45 | Rat | CD45 (leukocytes) | BD |
| Anti Glut-1 | Goat | Endoth. glucose transporter-1 | Santa Cruz Biotechnology, Dallas, TX |
| Anti Iba-1 | Rabbit | Activated microglia | Wako Pure Chemical Industries, Osaka, Japan |
| Anti-GFAP | Rabbit | Glial fibrillary acidic protein in astrocytes | Dako, Glostrup, Denmark |
| Anti-iNOS | Rabbit | iNOS | Santa Cruz Biotechnology |
| Anti β-APP | Rabbit | β-amyloid precursor protein, neurodegeneration | Zymed, San Francisco, CA |
| Anti-Chondroitin sulphate proteoglycan NG2 | Rabbit | Pericytes | Millipore |
| Anti-ZO-1 | Rabbit | Tight junction zona occludens-1 | Invitrogen, Carlsbad, CA |
| Anti-claudin-5 | Mouse | Tight junction claudin 5 | Invitrogen |
| Anti-occludin | Mouse | Tight junction occludin | Invitrogen |
| Anti-NF-κB p65 | Rabbit | NF-κB p65/ RelA | Santa Cruz Biotechnology |
| Anti-fibrinogen | Rabbit | Fibrinogen | Dako |
| Anti-mouse IgG | Rabbit | IgG | Dako |
| Anti-p38 MAPK | Rabbit | MAP kinase-p38 | Cell Signaling Technology |
| Anti-p38 phospho MAPK | Rabbit | Anti phospho MAP Kinase (Tyr 180/182) | Cell Signaling Technology |
