# Supplementary material for: Nitric Oxide Protects against Infection-Induced Neuroinflammation by Preserving the Stability of the Blood-Brain Barrier
Source: PLoS Pathog. 2016 Feb 25;12(2):e1005442. doi: 10.1371/journal.ppat.1005442 (PMC4767601; doi:10.1371/journal.ppat.1005442)
Supplement: S3 Table — (DOCX) [file ppat.1005442.s010.docx]

| ***Gene*** | **ID** | **Forward primer** | **Reverse primer** |
| --- | --- | --- | --- |
| *nos2* | NM_010927.3 | CAG CTG GGC TGT ACA AAC CTT | CAT TGG AAG TGA AGC GTT TCG |
| *hprt* | J00423.1 | CCC AGC GTC GTG ATT AGC | GGA ATA AAC ACT TTT TCC AAA TCC |
| *tnf* | NM_013693.3 | GGC TGC CCC GAC TAC GT | GAC TTT CTC CTG GTA TGA GAT AGC AAA |
| *mmp2* | NM_008610.2 | GTT GCT TTT GTA TGC CCT TCG | TCA GAC AAC CCG AGT CCT TTG |
| *mmp3* | NM_010809.1 | TCC TGA TGT TGG TGG CTT CA | TCC TGT AGG TGA TGT GGG ATT TC |
| *mmp12* | NM_008605.3 | TGT GGA GTG CCC GAT GTA CA | AGT GAG GTA CCG CTT CAT CCA T |
| *mmp8* | NM_008611.4 | GAT GGA CCC AAT GGA ATC CTT | TTC TTC TGA ATC AAA ATG AGC ATC TC |
| *mmp9* | NM_013599.3 | AAA ACC TCC AAC CTC ACG GA | GCT TCT CTC CCA TCA TCT GGG |
| *vcam1* | NM_011693.3 | GTG ACT CCA TGG CCC TCA CTT | CGT CCT CAC CTT CGC GTT TA |
| *icam1* | NM_010493.2 | CAA TTT CTC ATG CCG CAC AG | CTG GAA GAT CGA AAG TCC GG |
| *e-selectin* | M87862.1 | CCC TGC CCA CGG TAT CAG | ACG TGC ATG TCG TGT TCCA |
| *il17a* | NM_010552.3 | CCG CAA TGA AGA CCC TGA TAG | TCA TGT GGT GGT CCA GCT TTC |
| *cldn5* | NM_013805.4 | ACT GCC GCG AAC AGT TCC TA | TCC AGC TGC CCT TTC AGG T |
| *ocln* | NM_008756.2 | AGG ACG GAC CCT GAC CAC TA | GGT GGA TAT TCC CTG ACC CAG |
| *iba1* | D86382.1 | CAC AAG AGG CCA ACT GGT CC | GGG CAG CTC GGA GAT AGC TT |
| *ifng* | NM_008337.3 | GCT TTG CAG CTC TTC CTC AT | CAC ATC TAT GCC ACT TGA GTT AAA ATA GT |
| *il1b* | NM_008361.3 | TGG TGT GTG ACG TTC CCA TT | CAG CAC GAG GCT TTT TTG TTG |
| *il6* | NM_031168.1 | ACA AGT CGG AGG CTT AAT TAC ACA T | TTG CCA TTG CAC AAC TCT TTT C |
| *Cxcl10* | NM_021274.2 | GCT GCC GTC ATT TTC TGC | TCT CAC TGG CCC GTC ATC |
| *Ccl2* | NM_011333.3 | CATCCACGTGTTGGCTCA | GATCATCTTGCTGGTGAATGAGT |
| *Cxcl9* | NM_008599.4 | CTT TTC CTC TTG GGC ATC AT | GCA TCG TGC ATT CCT TAT CA |
