# Supplementary material for: Nitric Oxide Protects against Infection-Induced Neuroinflammation by Preserving the Stability of the Blood-Brain Barrier
Source: PLoS Pathog. 2016 Feb 25;12(2):e1005442. doi: 10.1371/journal.ppat.1005442 (PMC4767601; doi:10.1371/journal.ppat.1005442)
Supplement: S1 Text — (DOCX) [file ppat.1005442.s011.docx]

**Supporting experimental procedures**

### In vitro toxicity assay

Suspensions of *T. b. brucei* 6000 bloodstream *Tbb* forms/well or mammalian cells for MOLT4 5000 cells/well and L-929 10,000 cells/well were prepared and incubated with different concentrations of SNAP or GSNO in IMDM and 10% FCS at 37°C for 72 hours. 10 µl WST-1 reagent (Roche, Basel, Switzerland) was then added and the plates were further incubated for 2h at 37°C. Actively proliferating cells convert WST-1 reagent to formazan, which can be spectrophotometrically detected at 450 nm. The absorbance was recorded from the plates using a multi-well scanning spectrophotometer at an exciting wavelength of 450nm. The IC_50_ for each compound was calculated using Prism 6, Graph Pad software.

### S1 Table. Toxicity of NO donors SNAP and GSNO on T. brucei and mammalian cell lines

|  | IC_50_ (µM) | |
| --- | --- | --- |
|  | SNAP | GSNO |
| *T.b. brucei* | 79.6 | 534 |
| Raw | 184 | 558 |
| Raji | 151 | 115 |
| MOLT 4 | 43 | 580 |
| TM29 | 990 | 2000 |
| HL-60 | 86 | 121 |
| GT-1 | 117 | 990 |

Parasites and mammalian cell lines were incubated with serial dilutions of SNAP (S-nitroso-N acetylpenicillamine) or GSNO (S-nitrosoglutathione)). The IC_50_ was determined 72h after incubation with the compounds.

### S2 Table. List of specific antibodies used for immunohistochemistry

| **Antibodies** | **Species** | **Target** | **Source** |
| --- | --- | --- | --- |
| An Tat 1.1 VSG | Rabbit | *T. brucei* | ITG, Antwerp |
| Anti-CD4 | Rat | CD4 | BD Biosciences, Franklin Lakes, NJ |
| Anti-CD8 | Rat | CD8 | BD |
| Anti-CD45 | Rat | CD45 (leukocytes) | BD |
| Anti Glut-1 | Goat | Endoth. glucose transporter-1 | Santa Cruz Biotechnology, Dallas, TX |
| Anti Iba-1 | Rabbit | Activated microglia | Wako Pure Chemical Industries, Osaka, Japan |
| Anti-GFAP | Rabbit | Glial fibrillary acidic protein in astrocytes | Dako, Glostrup, Denmark |
| Anti-iNOS | Rabbit | iNOS | Santa Cruz Biotechnology |
| Anti β-APP | Rabbit | β-amyloid precursor protein, neurodegeneration | Zymed, San Francisco, CA |
| Anti-Chondroitin sulphate proteoglycan NG2 | Rabbit | Pericytes | Millipore |
| Anti-ZO-1 | Rabbit | Tight junction zona occludens-1 | Invitrogen, Carlsbad, CA |
| Anti-claudin-5 | Mouse | Tight junction claudin 5 | Invitrogen |
| Anti-occludin | Mouse | Tight junction occludin | Invitrogen |
| Anti-NF-κB p65 | Rabbit | NF-κB p65/ RelA | Santa Cruz Biotechnology |
| Anti-fibrinogen | Rabbit | Fibrinogen | Dako |
| Anti-mouse IgG | Rabbit | IgG | Dako |

### S3 Table. List of primer sequences used

| ***Gene*** | **ID** | **Forward primer** | **Reverse primer** |
| --- | --- | --- | --- |
| *nos2* | NM_010927.3 | CAG CTG GGC TGT ACA AAC CTT | CAT TGG AAG TGA AGC GTT TCG |
| *hprt* | J00423.1 | CCC AGC GTC GTG ATT AGC | GGA ATA AAC ACT TTT TCC AAA TCC |
| *tnf* | NM_013693.3 | GGC TGC CCC GAC TAC GT | GAC TTT CTC CTG GTA TGA GAT AGC AAA |
| *mmp2* | NM_008610.2 | GTT GCT TTT GTA TGC CCT TCG | TCA GAC AAC CCG AGT CCT TTG |
| *mmp3* | NM_010809.1 | TCC TGA TGT TGG TGG CTT CA | TCC TGT AGG TGA TGT GGG ATT TC |
| *mmp12* | NM_008605.3 | TGT GGA GTG CCC GAT GTA CA | AGT GAG GTA CCG CTT CAT CCA T |
| *mmp8* | NM_008611.4 | GAT GGA CCC AAT GGA ATC CTT | TTC TTC TGA ATC AAA ATG AGC ATC TC |
| *mmp9* | NM_013599.3 | AAA ACC TCC AAC CTC ACG GA | GCT TCT CTC CCA TCA TCT GGG |
| *vcam1* | NM_011693.3 | GTG ACT CCA TGG CCC TCA CTT | CGT CCT CAC CTT CGC GTT TA |
| *icam1* | NM_010493.2 | CAA TTT CTC ATG CCG CAC AG | CTG GAA GAT CGA AAG TCC GG |
| *e-selectin* | M87862.1 | CCC TGC CCA CGG TAT CAG | ACG TGC ATG TCG TGT TCCA |
| *il17a* | NM_010552.3 | CCG CAA TGA AGA CCC TGA TAG | TCA TGT GGT GGT CCA GCT TTC |
| *cldn5* | NM_013805.4 | ACT GCC GCG AAC AGT TCC TA | TCC AGC TGC CCT TTC AGG T |
| *ocln* | NM_008756.2 | AGG ACG GAC CCT GAC CAC TA | GGT GGA TAT TCC CTG ACC CAG |
| *iba1* | D86382.1 | CAC AAG AGG CCA ACT GGT CC | GGG CAG CTC GGA GAT AGC TT |
| *ifng* | NM_008337.3 | GCT TTG CAG CTC TTC CTC AT | CAC ATC TAT GCC ACT TGA GTT AAA ATA GT |
| *il1b* | NM_008361.3 | TGG TGT GTG ACG TTC CCA TT | CAG CAC GAG GCT TTT TTG TTG |
| *il6* | NM_031168.1 | ACA AGT CGG AGG CTT AAT TAC ACA T | TTG CCA TTG CAC AAC TCT TTT C |
